# The curious case of selenium hyperaccumulation in *Coelospermum decipiens* from the Cape York Peninsula (Queensland, Australia)

**DOI:** 10.1093/aob/mcae103

**Published:** 2024-06-25

**Authors:** Maggie-Anne Harvey, Katherine Pinto Irish, Hugh H Harris, Peter D Erskine, Antony van der Ent

**Affiliations:** Laboratory of Genetics, Wageningen University and Research, Wageningen, 6708 PB, The Netherlands; Centre for Mined Land Rehabilitation, Sustainable Minerals Institute, The University of Queensland, St Lucia, 4072, Australia; Centre for Mined Land Rehabilitation, Sustainable Minerals Institute, The University of Queensland, St Lucia, 4072, Australia; Department of Chemistry, The University of Adelaide, Adelaide, 5005, Australia; Centre for Mined Land Rehabilitation, Sustainable Minerals Institute, The University of Queensland, St Lucia, 4072, Australia; Laboratory of Genetics, Wageningen University and Research, Wageningen, 6708 PB, The Netherlands; Centre for Mined Land Rehabilitation, Sustainable Minerals Institute, The University of Queensland, St Lucia, 4072, Australia

**Keywords:** *Coelospermum decipiens*, hyperaccumulator, selenium, Cape York

## Abstract

**Background and Aims:**

The tropical shrub *Coelospermum decipiens* (Rubiaceae) is an extreme selenium (Se) hyperaccumulator, reported to accumulate up to 1140 µg Se g^−1^ when found growing on soils with levels of Se below the limit of detection (i.e. <0.01 mg Se kg^−1^) leading to a bioconcentration factor of >100 000.

**Methods:**

*Coelospermum decipiens* plants were sampled from different populations in far north Queensland and analysed for Se concentrations. Plant material was subjected to synchrotron X-ray fluorescence microscopy (XFM) and synchrotron X-ray absorption spectroscopy (XAS) investigations to gain insights into the elemental distribution and chemical speciation of Se.

**Results:**

The foliar Se concentrations ranged from 100 to 1000 µg Se g^−1^, except for the seeds, which had up to 28 000 µg Se g^−1^. The soils from the Hope Vale area were locally Se-enriched up to 48 mg Se kg^−1^, but there was no relationship between soil and plant Se concentrations. Synchrotron XFM analysis revealed that Se was localized in the blade margin tissue of the younger leaves, whilst the XAS analysis determined that Se occurs as an organo-Se compound.

**Conclusions:**

We report the occurrence of seleniferous soils in the Cape York Peninsula soils for the first time, which may partly explain the evolution of Se hyperaccumulation in *C. decipiens*. The extremely high concentrations of Se in the seeds is suggestive of a herbivory protection function. The capacity of this species to accumulate and hyperaccumulate Se from non-seleniferous soils is akin to that of other ‘seed’-based accumulators, such as some members of the Lecythidaceae family.

## INTRODUCTION

Selenium (Se), an essential element in the nutrition of animals, presents an interesting conundrum regarding its toxicity as overconsumption of Se leads to toxicity syndromes (Schwarz and Foltz, 1957; Rotruck *et al*., 1973). Selenium toxicity (selenosis) has historically been reported in livestock in areas where seleniferous soils occur, such as in parts of the USA, China, India, Australia and Venezuela (Oldfield, 2002). Selenosis was widely recognized in the 1930s in the USA, when it was discovered that livestock grazed in areas of high-Se soil developed symptoms aligning with alkali disease, such as hoof lesions and hair loss (Beath *et al*., 1934; Draize and Beath, 1935; O’Toole *et al*., 1996). Chronic selenosis is caused by continuous ingestion of plants containing high Se (5–50 µg Se g^−1^), particularly grains and grasses (Rosenfeld and Beath, 1964). Most known Se hyperaccumulators occur exclusively on seleniferous soils, such as those in Wyoming or north-west Queensland. The work of O. A. Beath, H. G. Byers and co-workers on the plants of seleniferous areas of the USA in the 1930s and 1940s has been briefly summarized by Reeves and Baker (2000), who give a list of 20 species (in seven different families) from the USA, Australia and Venezuela that were recorded with Se concentrations >1000 µg Se g^−1^ DW (Reeves and Baker, 2000). More recently, attributes of all known Se hyperaccumulators have been summarized in a review by White (2016). In the seleniferous areas of the western USA, plants implicated in cattle selenosis have been extensively studied, such as *Astragalus racemosus* and *A. bisulcatus* (Fabaceae) containing 2500–15 000 µg Se g^−1^, and *Stanleya pinnata* (Brassicaceae) containing >4000 μg Se g^−1^ (Byers, 1935; Rosenfeld and Beath, 1964; Freeman *et al*., 2012; Cappa *et al*., 2014; Statwick, 2016; Lima *et al*., 2022). *Neptunia amplexicaulis* (Fabaceae), responsible for selenosis cases in the Richmond region of north-west Queensland, can accumulate up to 13 600 µg Se g^−1^ in its leaves in experimental conditions and 4334 µg Se g^−1^ in the field (Knott and McCray, 1959; McCray and Hurwood, 1963; Harvey *et al*., 2020). Selenium is considered a beneficial, but not an essential, element in plants, although it can confer increased tolerance to environmental factors and provides resistance to pathogens and herbivory (Hartikainen, 2005; Quinn *et al*., 2008, 2010; Pilon-Smits *et al*., 2009; Zhang and Gladyshev, 2010; El Mehdawi and Pilon-Smits, 2012; Feng *et al*., 2013). Whether Se is essential in some obligate Se hyperaccumulator plant species is open to debate, although there has been some genetic evidence for Se essentialty in some algae (Fu *et al*., 2002; Lobanov *et al*., 2007; Sors *et al*., 2009).

### Seleniferous soils and the ecophysiology of selenium hyperaccumulators

Typically, soils contain somewhere between 0.5 and 2 µg Se g^−1^, but seleniferous soils can contain >2 to >100 µg Se g^−1^, often when derived from marine shales and limestones, or in areas polluted by the disposal of fly-ash or saline irrigation wastewater (Rosenfeld and Beath, 1964; Kushwaha *et al*., 2022). Seleniferous areas in Queensland were recorded with up to 69 µg Se g^−1^ in the most poisonous areas (Knott and McCray, 1959). Most soluble forms of Se, selenate (SeO_4_^2−^) and selenite (SeO_3_^2−^) are often taken up by plants through sulphate pathways and phosphate pathways respectively, and either kept in inorganic forms or transformed into seleno-amino acids, particularly in the case of Se hyperaccumulators (Anderson, 1993; Hopper and Parker, 1999). For example, *N. amplexicaulis* synthesizes selenocystathionine (SeCT), whereas *A. bisulcatus* has been found to primarily contain methylselenocysteine (MeSeCys) (Peterson and Butler, 1967; Harvey *et al*., 2020). Overexposure to Se in non-tolerant plants can lead to reduced growth and toxicity due to seleno-protein overproduction and oxidative stress from inorganic Se accumulation (Van Hoewyk *et al*., 2008; Van Hoewyk, 2013). The distribution of Se in the plant differs between hyperaccumulators, but with some recurring patterns. X-ray fluorescence microscopy of hyperaccumulator plant tissues using laboratory and synchrotron methods has revealed that Se tends to be sequestered in the epidermal cells, in the pulvini and around the vascular tissue of *Astragalus* species, in the marginal leaf tissues of *S. pinnata*, and in the vascular tissues and young leaves of *N. amplexicaulis* (Pickering *et al*., 2003; Freeman *et al*., 2006*b*; Harvey *et al*., 2020; van der Ent *et al*., 2023). All species analysed for Se distribution contain highly concentrated Se in the reproductive tissues and seeds (Quinn *et al*., 2011; Lima *et al*., 2018). The paradise nut tree (or ‘coco de mono’) of Venezuela, *Lecythis ollaria* (Lecythidaceae), can contain up to 12 000 µg Se g^−1^ in the nut, which is therefore also highly toxic (Hammel *et al*., 1996; Müller and Desel, 2010). Among the consumable nuts, *Bertholletia excelsa* (Lecythidaceae), or Brazil nut, is a notable source of additional Se where required in the human diet as it has been found with highly variable Se concentrations of <0.03–512 µg Se g^−1^ in fresh nuts from Brazil (Chang *et al*., 1995). Conversely, commercially available Brazil nuts yielded 28–49 µg Se g^−1^, and other fresh nuts from South America contained only 2–20 µg Se g^−1^; concentrations in the dry matter or in defatted material would be somewhat higher (Parekh *et al*., 2008; Lima *et al*., 2019). *Bertholletia excelsa*, notably, has not been found growing on exceedingly seleniferous soils, though concentrations found in the trunk and leaf tissues correlated with varying concentrations of Se in the soil (Silva Junior *et al*., 2017; Castro *et al*., 2019).

### *The curious case of* Coelospermum *decipiens*

The *Coelospermum* genus is found from southern China to Indochina and the western Pacific, and one interesting species in this genus, *Coelospermum decipiens* (Rubiaceae; formerly *Morinda reticulata*), occurs in New Guinea to north Queensland (Zich *et al*., 2020). It is locally called Mapoon bush and the roots were used by the Aboriginal people of Cape York as a yellow dye source or as a contraceptive (Langevad, 1983). Chronic selenosis in Australia has been described in horses and cattle from north-western Queensland and in horses from the Cape York Peninsula (Knott *et al*., 1958). The Cape York Peninsula had cases of ‘change hoof disease’ since its settlement in 1864, and vegetation surveys matched the spatial distribution of the disease to the distribution of *C. decipiens* and its quick resprouting following pasture burning and monsoonal rains (Knott and McCray, 1959). Most *C. decipiens* leaves were found to contain around 200 µg Se g^−1^, with values reaching up to 1141 µg Se g^−1^ (Knott and McCray, 1959). As this plant can accumulate over 1000 µg Se g^−1^, it is classified as a Se hyperaccumulator, but unlike many other plants with elevated Se these plants were reported growing in soils with very low Se status, containing <0.01 µg Se g^−1^ (Knott and McCray, 1959; White, 2016). *Coelospermum decipiens* is the only Se hyperaccumulator in the Rubiaceae family and the only known Se hyperaccumulator recorded from low-Se soil. This may be explained by either incorrect soil analysis or cryptically seleniferous soils in *C. decipiens* habitat in Queensland, or it might pose a unique modality of Se hyperaccumulation. The species has been shown to contain SeCT in its leaf tissues; the same compound was discovered in *N. amplexicaulis* using the same analytical methods (Peterson and Butler, 1967, 1971). Ethanol-soluble fractions were extracted and analysed with paper chromatography and electrophoresis, which, while successful at identifying Se compounds, is limited in investigating inorganic forms of Se in comparison with modern X-ray absorption spectroscopy (XAS) (Weekley *et al*., 2013; van der Ent *et al*., 2018). The original field surveys conducted in the late 1950s discussed the need for informed land management to avoid selenosis in cattle (Knott and McCray, 1959). With no new recorded cases of selenosis in the region, no new research on Se hyperaccumulation in *C. decipiens* has been published in the last 50 years, despite its unusual relationship with Se hyperaccumulation in soils with putative low Se status. Furthermore, no assessment of Se uptake capacity or Se tissue-level distribution or analysis of Se chemical speciation using modern techniques has been undertaken for this species specifically.

This study aimed to determine the chemical forms and distribution of Se in *C. decipiens*, as well as to determine the Se accumulation capacity in plants from the natural habitat and in plants in Se dosing treatments under controlled conditions. To that end, we sampled plants from near Hope Vale in far north Queensland and propagated plant material from Weipa for a dosing experiment. This was combined with elemental analysis and microanalytical characterization of Se chemical form and tissue-level distribution using X-ray analytical techniques, including synchrotron X-ray fluorescence microscopy (XFM) and XAS. Furthermore, herbarium specimens of the *Morindeae* tribe of Rubiaceae, including the genera *Morinda, Coelospermum* and *Gynochthodes*, were screened for the possible occurrence of Se hyperaccumulation to provide insight into phylogenetic patterns of Se hyperaccumulation in this family.

## MATERIALS AND METHODS

### *Herbarium X-ray fluorescence measurement of selenium in* Coelospermum *specimens*

The use of handheld X-ray fluorescence (XRF) instruments is a non-destructive and effective method for the systematic quantitative assessment of hyperaccumulation in vast numbers of herbarium specimens (van der Ent *et al*., 2019). The XRF analyser (ThermoFisher Niton Xlt3-950) uses a miniaturized X-ray tube (Ag-target, 25–50 kV, 200 μA) as its main excitation source. An XRF analysis was undertaken at the Queensland Herbarium (BRI) in Brisbane on all available specimens of the Morindeae tribe of the Rubiaceae, which includes the genera *Morinda* (synonym *Pogonolobus*) (*n* = 2 taxa, 190 specimens), *Coelospermum* (*n* = 5 taxa, 511 specimens) and *Gynochthodes* (*n* = 10 taxa, 683 specimens). Each measurement was taken from specimens attached to standard herbarium cardboard sheet placed on top of a 100-cm^2^ 99.995 % pure titanium plate (to block transmitted X-rays and to provide a uniform background) and measured in soil mode for 30 s. Values below the limit of detection (LOD) threshold were excluded from the dataset. Soil dust contamination adhering to leaves can confound measurements, but may be gauged from unusually high concomitant Cr, Fe and Ti concentrations (Cary and Kubota, 1990) and suspect specimens were omitted from the dataset. Where distinguishable, both older and younger leaves were scanned.

### Plant propagation and selenium dosing experiment

Cuttings of *C. decipiens* were collected near Weipa (Queensland) and rooted in a mixture of perlite and vermiculite using Clonex Red rooting hormone (8.0 g L^−1^ indole-3-butyric acid; Growth Technology, Australia). After ~6 weeks the rooted cuttings were transferred to natural soil (Ferralsol) originating from Weipa. Plants were watered from the bottom when the soil became dry and maintained for several months to establish root systems and develop new leaves. Half of the surviving population were then watered with 100 mL of solution containing 5 mg Se L^−1^ soil in each pot, as sodium selenate (Na_2_SeO_4_), once per week for a period of 3 months. The remaining living specimens were collected (14 dosed and 14 non-dosed specimens), washed three times with deionized water to remove contaminating soil, and separated into young leaves, old leaves (if present), stems and roots. Specimens were dried for 48 h at 60 °C in a dehydrating oven.

### Field collections of plant tissue samples

**Fig. 1.**
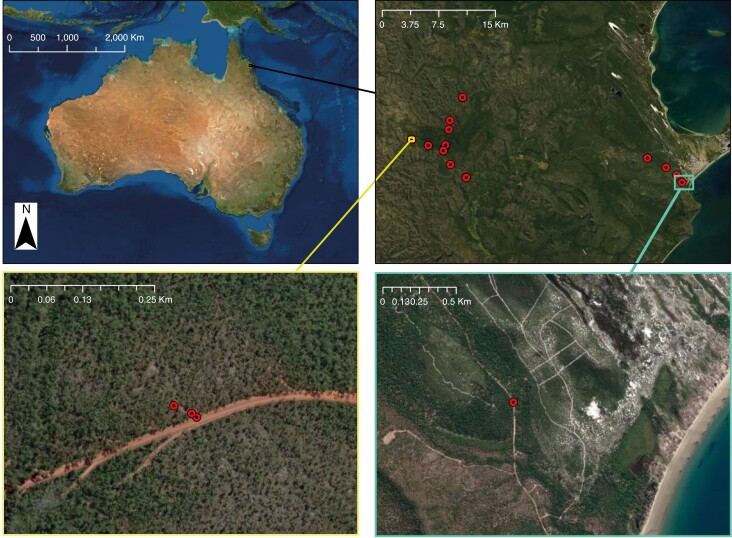
Clockwise from top left: map showing the Hope Vale area in relation to Australia; sampling locations (inland left, coastal right); sample 25 at Elim Beach locality; samples 4, 5 and 6 at the upper Endeavour Battlecamp locality.

**Fig. 2.**
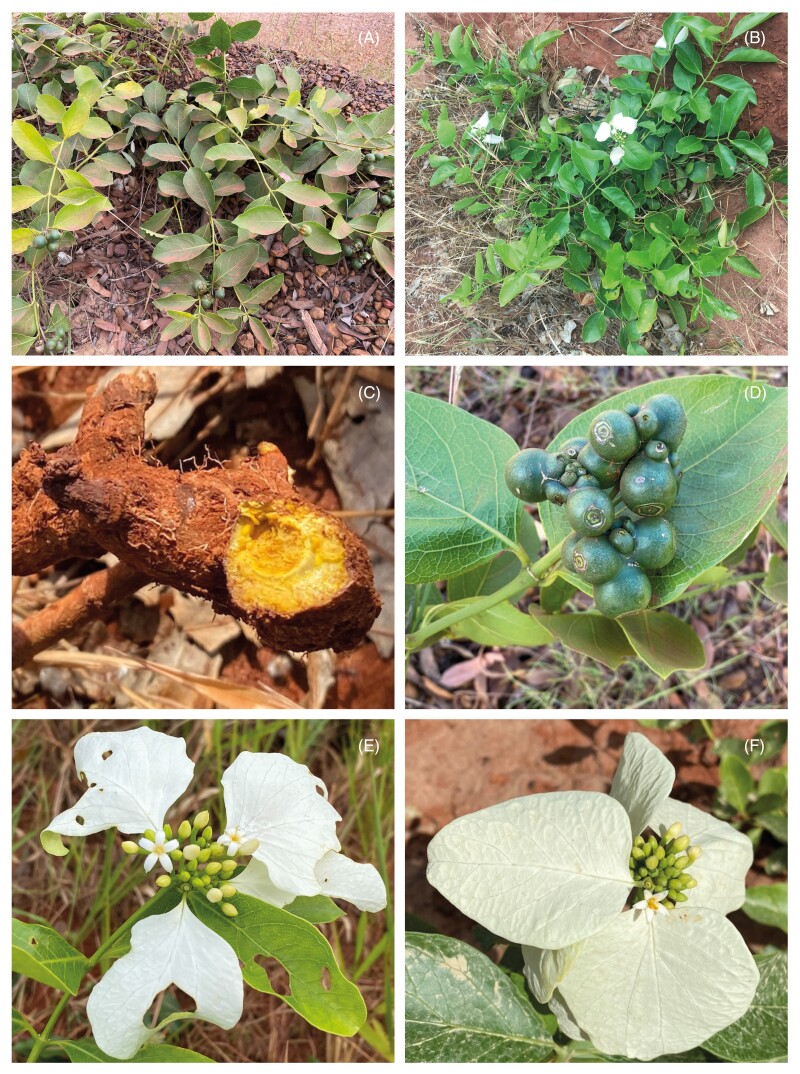
*Coelospermum decipiens* in the field near Hope Vale in Far North Queensland. (A) Plant with seeds. (B) Plant with flowers. (C) Root, cut side showing intense yellow pigment. (D) Fruit, seedpods (E) and (F) flowers and flower buds with floral bracts (white calycophylls).

A field expedition took place in June 2021 around the Cooktown and Hope Vale area. *Coelospermum decipiens* specimens were collected from inland and coastal areas, with 20 specimens collected from the inland locations along Endeavour Battlecamp Road and Isabella McIvor Road, and five coastal specimens collected at Elim Beach (Fig. 1; Supplementary Data Fig. S1). Plant samples collected included young leaves, old (basal) leaves, young stem, basal stem, and root tissue (Fig. 2). Where available, floral bracts, flower buds, flowers and fruit were also collected (Fig. 2). Samples were dried in an oven at 60 °C for 48 h.

### Bulk chemical analysis of plant tissue samples

Plant tissue samples were ground to a fine powder in an impact mill at 19 000 rpm (Tube Mill 100 control with disposable titanium blades) and weighed to 100 ± 5 mg in 6-mL polypropylene tubes. In the case of flowers and flower buds, samples were weighed up to 100 ± 5 mg directly, and fruits were separated into seed coat, mesocarp and seed before being manually weighed up to 100 ± 5 mg. These samples were pre-digested using 2 mL HNO_3_ (70 % v/v) for 24 h before being digested in a block heater (Thermo Scientific™ digital dry bath) for a 2-h programme (1 h at 70 °C followed by 1 h at 125 °C) and diluted to 10 mL with ultrapure water (Millipore 18.2 MΩ cm at 25 °C) before analysis with inductively coupled plasma atomic emission spectroscopy (ICP–AES) with a Thermo Scientific iCAP 7400 instrument for macro-elements (Na, Mg, Al, P, S, K, Ca), trace elements (Cr, Mn, Fe, Co, Ni, Cu, Zn) and ultra-trace elements (As, Se, Cd, Tl) in radial and axial modes depending on the element and expected analyte concentration. All elements were calibrated with a four-point curve covering analyte ranges in the samples. In-line internal addition standardization using yttrium was used to compensate for matrix-effect interferences. Quality controls included matrix blanks and standard reference material (NIST Apple Leaves 1515).

### Collection and chemical analysis of soil samples

Soil was collected from 5 to 20 cm depth, directly around the root zone of field samples, air-dried to constant weight and sieved through a 2-mm screen. Soil sub-samples were weighed to 100 ± 5 mg in quartz digestion vessels and 5 mL HNO_3_ (70 %) and 2 mL HCl (37 %) were added. The samples were then digested for 16 min at 50 % power using a ColdBlock system (CB15S 15 channel system; ColdBlock Technologies), which uses high-intensity infrared irradiation to aid rapid acid digestion (Wang *et al*., 2014). The digestates were quantitatively transferred to 50-mL tubes, brought to volume (40 mL), and then filtered (Whatman^®^ Grade 41 filter paper) before analysis with ICP–AES. Plant available Se was extracted using the AB–DTPA method (Soltanpour and Schwab, 1977; Sharmasarkar and Vance, 1995), which uses 1 M ammonium bicarbonate and 0.005 M DTPA (diethylenetriaminepentaacetic acid). The AB–DTPA solution was prepared by dissolving 1.97 g of DTPA in 800 mL distilled water to which 2 mL of 1:1 NH_4_OH was added. Then 79.06 g NH_4_HCO_3_ was added and dissolved, and the pH adjusted to 7.6 using NH_4_OH or HCl and brought to volume (1.0 L). Ten grams of soil was extracted with 20 mL AB–DTPA solution for 30 min on a reciprocating shaker, then centrifuged (Eppendorf Centrifuge 5810, 10 min at 3220 x *g*), filtered (Whatman^®^ Grade 41 filter paper) and 9.5 mL was pipetted into 10-mL tubes. Finally, 0.5 mL of HNO_3_ (70 %) was added to drive off CO_2_ before analysis with ICP–AES as described above. In addition, Sr(NO_3_)_2_-extraction (0.01 m) was performed using a method adapted from Kukier and Chaney to determine weakly exchangeable metal(loid) concentrations in the soil (solid/liquid ratio, m:v, of 1:4 for 2 h) (Kukier and Chaney, 2001).

### Synchrotron X-ray fluorescence *microscopy*

The XFM beamline at the Australian Synchrotron employs an Si(111) monochromator and a pair of Kirkpatrick–Baez mirrors to deliver X-rays onto the specimen with fluorescent X-rays collected in backscatter geometry using the 384-element Maia detector system (Kirkham *et al*., 2010; Ryan *et al*., 2010; Lombi *et al*., 2011; Paterson *et al*., 2011). The possibility of radiation-induced damage in XFM analysis (especially in fresh hydrated samples) is an important consideration that may limit the information sought from the analysis (van der Ent *et al*., 2018). In a recent study, radiation dose limits for XFM analysis were assessed, and in hydrated plant tissue dose limits are 4.1 kGy before detectable damage occurs (Jones *et al*., 2019). In order to limit radiation damage, we used fast scanning (per-pixel dwell time is <10 ms), hence the effective radiation dose is low. Leaf, stem and root tissues from glasshouse-grown, Se-dosed cuttings were held between two sheets of polyethylene (Ultralene) thin film (4 μm) stretched over a Perspex frame to prevent dehydration duration the measurement.

### Synchrotron X-ray absorption spectroscopy

Crushed leaf tissues from dosed propagated specimens of *C. decipiens* were sealed in Kapton tape and cooled to ~5 K in an He expansion cryostat. The XAS spectra of the samples were recorded in fluorescence mode. The energy ranges utilized for XANES Se K-edge data collection are pre-edge region 12 430–12 635 eV (10-eV steps); XANES region 12 635–12 685 eV (0.25-eV steps); and post-edge region 12 685–12 875 eV. A spectrum of a hexagonal Se standard, recorded simultaneously in transmission downstream of the sample, was used to calibrate the energy scale to the first peak of the first derivative of the elemental Se edge (12 658.0 eV). Spectra of model selenium compounds for XANES linear combination fitting are elemental selenium (red Se allotrope), Na_2_SeO_4_, Na_2_SeO_3_, seleno-l-cystine, seleno-l-methionine, and Se-(methyl)selenocysteine. The red Se allotrope of elemental selenium was synthesized by reduction in a solution of Na_2_SeO_3_ with excess ascorbic acid.

### Data analysis

The XRF event stream was analysed using the dynamic analysis method as implemented in GeoPIXE (Ryan *et al*., 1990, 2005; Ryan and Jamieson, 1993; Ryan, 2000). This method generates elemental images, which are (1) overlap-resolved, (2) with subtracted background and (3) quantitative, i.e. in units of micrograms per gram dry weight. The XAS data were interpreted with standard approaches using EXAFSPAK. All data were calibrated, background-corrected and normalized and the XANES spectra were compared with spectra of a range of Se compounds that act as model compounds by deconvolution of spectra using principal component analysis and multiple linear regression statistical techniques. Statistical analyses were performed using R version 4.0.2 and RStudio version 2022.02.2 + 485 (Integrated Development for R; RStudio, PBC, Boston, MA, http://www.rstudio.com) and Microsoft Excel 2016 (Redmond). The Se concentrations of wild and dosed specimen tissue biomass (g) are presented as boxplots (R package ggplot2) and in tables. The mean and standard deviation were determined using the R package rstatix and significant differences were tested using non-parametric one-way ANOVA (Kruskal–Wallis test) with confidence level 95 % and the *post hoc* pairwise Wilcoxon rank sum test (with Bonferroni adjustment) in RStudio. The relationship between soil and plant Se concentrations was determined using simple linear regression analysis in RStudio, with plant Se data undergoing log transformation to meet test assumptions.

## RESULTS

### Systematic assessment of incidence of selenium accumulation in the Morindeae tribe

From herbarium XRF scanning, only *C. decipiens* in the Morindeae tribe had >15 µg Se g^−1^, and average Se concentrations ranged from below the LOD to 3 µg Se g^−1^ for all other species (Supplementary Data Table S1). *Coelospermum decipiens* had 72 µg Se g^−1^ on average, with some young leaves reaching up to 639 µg Se g^−1^, which was statistically different from the distributions of every other species measured in this tribe (*P* < 0.05) except for *Gynochthodes australiensis*, which was due to the very limited sample size for *G. australiensis* (*n* = 2, *P* = 0.32). There was no significant difference in the Se concentrations between young and old leaves within a given species (*P* = 0.66).

### Selenium concentrations in Hope Vale and Cooktown soils

Soils taken from near the root zones of all Hope Vale specimens contained varying concentrations of Se, ranging from below the LOD to 49 mg Se kg^−1^ (Table 1; Supplementary Data Table S2). The soils from the Elim Beach localities had no detectable Se, with the exception of a single sample (sample 24), which contained 16 mg Se kg^−1^ of total recoverable Se. Of the extractable (bioavailable) Se using the AB–DTPA method, the only soil from Elim Beach that had detectable Se (sample 25) contained 0.122 mg Se kg^−1^. The soil samples were white–red quartz sand, derived from underlying Hodgkinson Formation cherts and quaternary sandstones and silts (Domagala *et al*., 1993, 1997). Inland samples along Endeavour Battlecamp Road and Isabella McIvor Road were a variety of sandy and distinctly ferruginous soils derived from the Gilbert River Formation sandstones, Dalrymple sandstones and Quaternary weathered silts and sands, and some inclusion from the Piebald Basalts and ferricrete/ferruginous duricrusts (Lucas and de Keyser, 1965; Smart *et al*., 1972; Domagala *et al*., 1997). On Endeavour Battlecamp Road, the grouping of samples 2–7 had a variety of total Se concentrations between not detectable to 18 mg Se kg^−1^; the grouping of samples 8–11 had soils that ranged from 17 to 33 mg Se kg^−1^. The Isabella McIvor Road locality had total Se concentrations from not detectable to up to 26 mg Se kg^−1^; however, two samples (18 and 19) from around the ferruginous duricrust outcrop contained 43.5–48.7 mg Se kg^−1^ total. Bioavailable Se, however, only reached a maximum of 0.50 mg Se kg^−1^ at locality 13 and averaged 0.087 mg Se kg^−1^ across the Isabella McIvor localities and 0.028 mg Se kg^−1^ across the Endeavour Battlecamp locality, with localities 18 and 19 only containing bioavailable Se below the LOD and 0.007 mg kg^−1^, respectively (Table 1).

**Table 1.** ColdBlock digest (total), AB–DTPA-extractable and strontium nitrate-extractable Se concentrations in soil from Hope Vale. Concentrations are given as generated through ICP–AES analysis, with the minimum, maximum and mean (in parentheses) values from each locality. Means were calculated using LOD/√2 to replace values below the LOD. The LOD of Se for each analysis is in the top row.

Locality	*n*			
		ColdBlock digest	AB-DTPA extraction	Strontium nitrate extraction
		**LOD (mg kg** ^**−1**^ **)**		
		5.57	0.005	0.014
		**Se (mg kg** ^**−1**^ **)**		
Endeavour Battlecamp	10	<LOD, 33.1 (15.7)	<LOD, 0.265 (0.048)	<LOD
McIvor River	9	<LOD, 48.7 (20.9)	<LOD, 0.50 (0.09)	<LOD
Elim Beach	5	<LOD, 16.0 (6.30)	<LOD, 0.12 (0.028)	<LOD

### *Selenium concentrations in* Coelospermum decipiens *in the natural habitat*

**Fig. 3.**
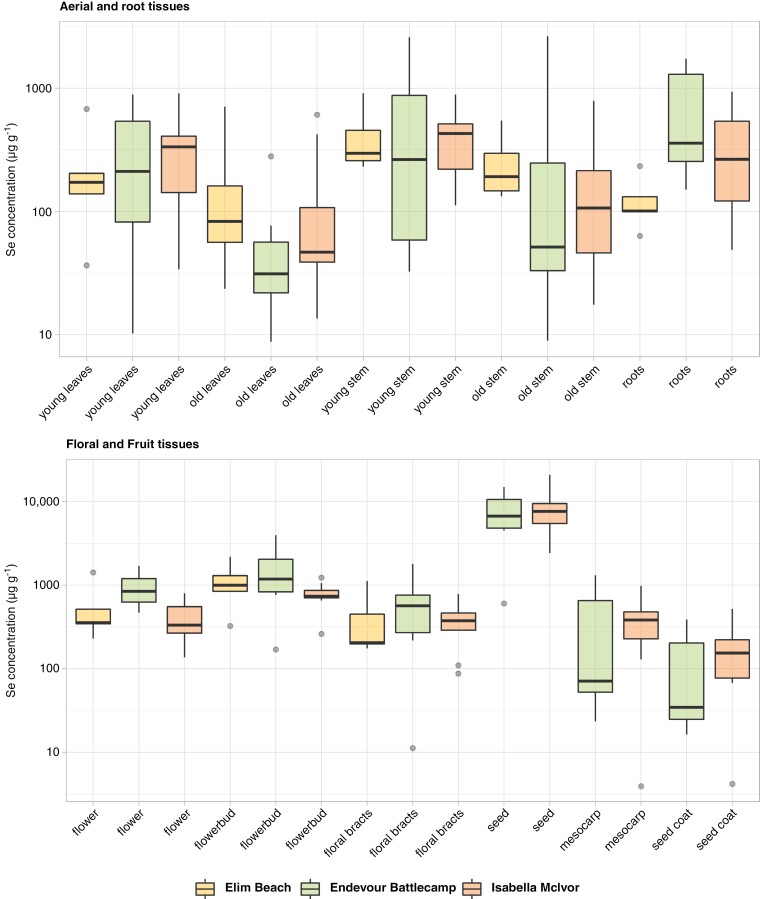
Boxplot showing Se concentrations (µg g^−1^) in *C. decipiens* tissues collected from Hope Vale, QLD measured with ICP–AES. Boxplots show median, range and outliers (circles), across different tissues and distinguishing three localities. Aerial and root tissues *n* = 24, flowers *n* = 15, floral bracts and flower buds *n* = 20, fruit tissues *n* = 16. Samples below the LOD were replaced with LOD/√2 in mg L^−1^ transferred to µg g^−1^ using original sample weight. The *Y* axis is presented on a log_10_ scale.

The seeds contained a higher Se concentration than all other tissues (mean 8116 and maximum 20 777 μg Se g^−1^, *P* < 0.0001), followed by the flower buds (mean 1159 μg Se g^−1^), which were significantly higher than all other tissues except the seeds (*P* < 0.025; Fig. 3; Supplementary Data Tables S3 and S4). The flowers (mean 585 μg Se g^−1^), in comparison, had a lower Se concentration than this (*P* = 0.024). Non-seed fruit tissues such as the mesocarp (mean 379 μg Se g^−1^) and seed coat (mean 155 μg Se g^−1^) were either similar to or significantly lower than most other tissues, except that the mesocarp had a significantly higher concentration of Se than the older leaves (*P* = 0.027). The leaf tissues had amongst the lowest Se concentrations of tissues overall; the young leaves (mean 316 μg Se g^−1^) were only significantly higher than the old leaves (mean 124 μg Se g^−1^; *P* = 0.004), and the older stems were also low in Se (mean 320 μg Se g^−1^; *P* < 0.04), statistically similar to the leaves and non-seed fruit tissues. The younger stems (mean 534 μg Se g^−1^), root tissues (mean 470 μg Se g^−1^) and floral bracts (mean 475 μg Se g^−1^) had statistically similar ranges, with significantly higher Se concentrations than the old leaves, old stems and seed coats (*P* < 0.05). It should be noted that the specimens from Elim beach had not produced seeds at the time of collection, so no seed material analysis is available for this population.

When compared within tissues, Se values were not significantly different between localities sampled, with the exception of the root tissues (Kruskal–Wallis test, *P* = 0.024), which had significantly lower Se in the coastal Elim beach specimens (mean 126 μg Se g^−1^) than the inland Endeavour Battlecamp specimens (mean 737 μg Se g^−1^; Wilcoxon’s test, *P* = 0.008). Similarly, when comparing Se tissue concentrations with the Se concentrations in the soil, there was very little significant correlation overall; however, the root Se concentration loosely correlated with the total soil Se concentration (*P* = 0.002; *R*^2^ = 0.35; Supplementary Data Figs S2 and S3). Bioavailable soil Se was not correlated with plant tissues overall (*P* > 0.1). Specimens from Elim Beach were flowering but had not yet produced seeds, and consequently no data are available for fruit tissues from this population; however, all floral tissue contained Se in concentrations indistinguishable from the other localities (*P* < 0.05).

Herbarium specimen AQ325702, collected inland from Lockhart River in Cape York in 1948, was measured for Se concentrations in the sectioned tissue. In this instance, Se in flower tissue measured 3343 μg Se g^−1^ and leaf tissue contained 1463, but the seed coat and seed only contained 350 and 260 μg Se g^−1^, respectively (Supplementary Data Table S5).

### *Selenium concentrations in* Coelospermum decipiens *from Weipa and dosing experiment*

The stock tissue used for the dosing trial was taken from the Weipa population. The highest concentration recorded in Weipa *C. decipiens* tissue was 565 µg Se g^−1^ in the seed tissue (Supplementary Data Tables S6 and S7; Supplementary Data Fig. S4). The Se concentrations in the seeds were statistically different from those in all other organs (*P* < 0.05). All other tissues were not statistically different from each other, though root tissues (maximum 270 μg Se g^−1^) appeared to be higher in Se than all leaf or stem tissues, and younger leaves (maximum 80 μg Se g^−1^) were comparable to seed coat tissues (*P* > 0.05). Cuttings from the Weipa stock material were selected for the experiment once they had established in soil and developed leaves (Supplementary Data Fig. S5). The highest value recorded (759 µg Se g^−1^; Supplementary Data Tables S8 and S9) for glasshouse-grown cuttings was in the root tissues of plants dosed with 5 mg Se L^−1^ solution. The root tissues were significantly more concentrated in Se than every other tissue (*P* < 0.001; Supplementary Data Table S10). There was no significant difference in the Se levels overall between treatments (*P* = 0.094). Interactions between treatments and plant tissues, using two-way ANOVA and a non-parametric Scheirer–Ray–Hare test, were non-significant (*P* = 0.079 and 0.891, respectively).

### *Elemental distribution in fresh hydrated tissues of* Coelospermum decipiens

**Fig. 4.**
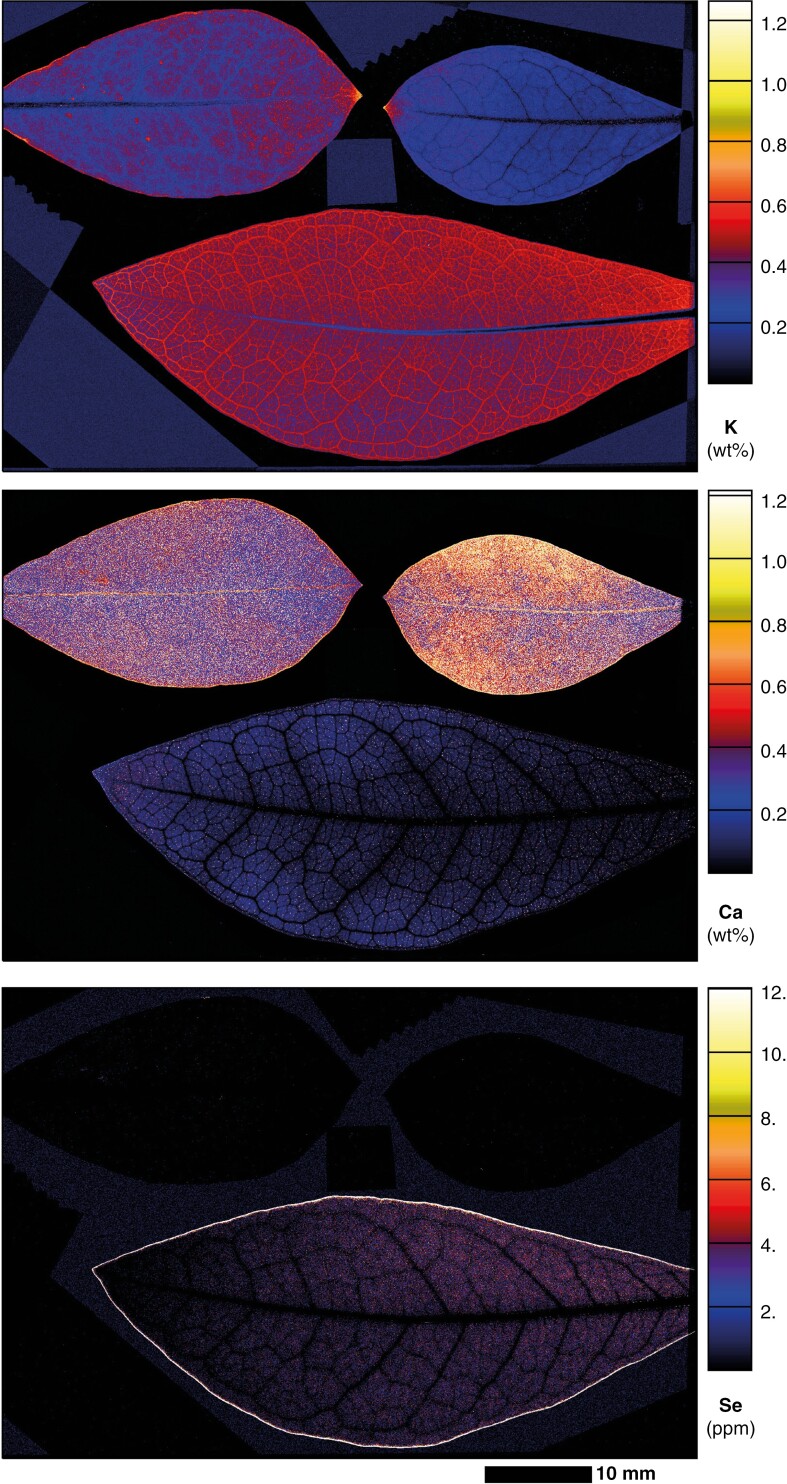
Synchrotron XFM maps of Ca, K and Se in hydrated excised whole old leaves (top) and young leaf (bottom) of *C. decipiens*.

**Fig. 5.**
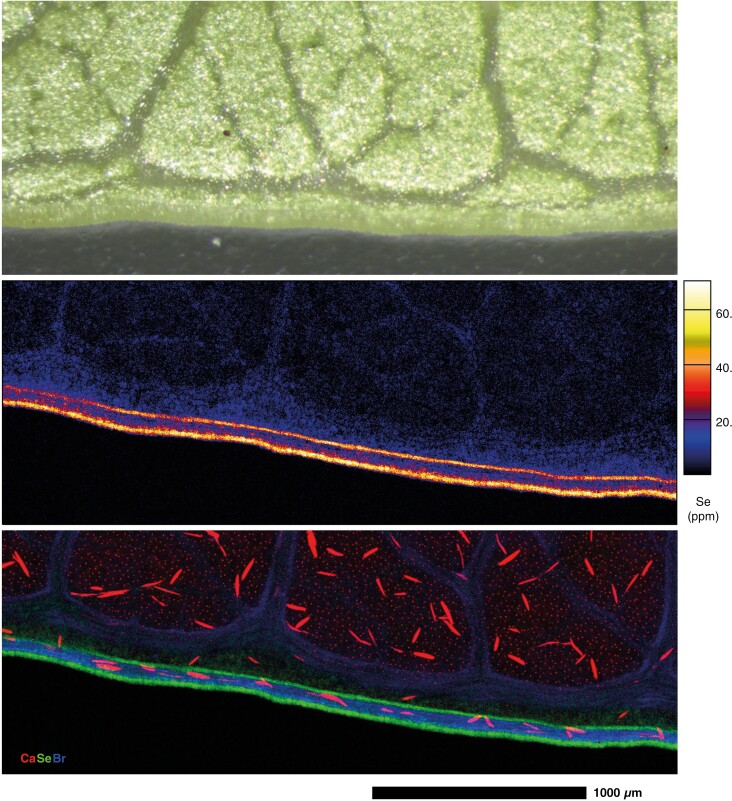
From top to bottom: light microscope image of hydrated young leaf margin portion of *C. decipiens.* Synchrotron XFM map showing Se concentration in leaf margin of *C.decipiens*. RGB Synchrotron XFM map showing Ca (red), Se (green) and Br (blue) in leaf margin of *C.decipiens*. A colour version of this figure appears in the online version of this article.

Leaves of glasshouse-cultivated Se-dosed *C. decipiens* plants grown from cuttings and analysed using synchrotron XFM revealed low prevailing Se distributed throughout the leaf lamina, with most Se concentrated in the blade margin (Fig. 4). Vascular tissues, including the marginal loop vein, were low in Se when compared with the blade margin, which does not appear vascular in nature, and appears on the borders of this marginal tissue (Fig. 5). The concentrations of Se are higher in the young leaf compared with the older leaves; this may be due to the younger leaf measured having developed during or after dosing with Se, whereas the older leaves may have developed fully prior to the start of dosing. In the young leaf, K is notably low from the midrib, and highest in the lateral and marginal veins and at medium–low concentrations in the lamina (Fig. 4). Potassium was localized at the apical end, at the margins, and within the lamina of the old leaves. The old leaves had a denser distribution of Ca oxalate crystals than younger leaves; the oldest leaf contained very high concentrations of Ca in the midrib and lamina, particularly at the margins, and the deposits were more apparent in the elemental maps. The younger leaves had far fewer crystals and the veins had no Ca, although examination of the intramarginal vein revealed prism-shaped crystals lining the marginal veins, whilst they were heterogeneously distributed in the lamina, and there were smaller homogeneously distributed deposits of Ca across the lamina surface (Fig. 4).

**Fig. 6.**
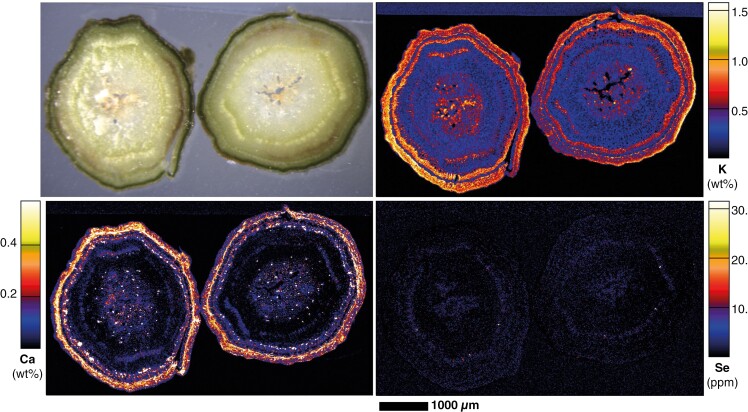
Light microscope image and synchrotron XRF maps of Ca, K and Se in hydrated stem cross-sections of *C. decipiens*.

**Fig. 7.**
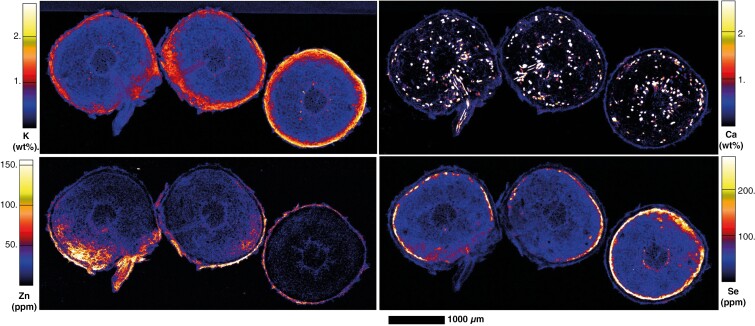
Synchrotron XRF maps of K, Ca, Zn and Se in hydrated root cross-sections of *C. decipiens.*

The XFM elemental maps of stem cross-sections show that Se is present at very low concentrations (<20 µg Se g^−1^), with spots of higher concentration in the xylem tissues (Fig. 6). Potassium is very high in the cortex, parts of the central pith and the procambium, and mostly present at low concentrations elsewhere. There were hotspots of high K nearer to the primary xylem. Calcium was located in the cortex and occurred in the phloem in lower concentrations with heterogeneously distributed high spots. Similarly, the inner pith had these high spots amongst overall low concentrations of Ca, and the xylem was mostly devoid of Ca. For young roots developed from cuttings, Se was present in phloem of the roots, but was only found in low concentrations in the secondary xylem and remnant cortex, which had high spots of Zn and Ca (Fig. 7). Potassium was found in the pericycle and phloem.

### *Chemical speciation of selenium in tissues of* Coelospermum decipiens

X-ray absorption spectroscopy at the Se K absorption edge was performed on *C. decipiens* tissues and the results of linear combination fitting of XANES (X-ray absorption near edge structure) spectra to a selection of model selenium compounds are shown in Table 2. All sample spectrum fits were dominated by a contribution from either the model Se-methyl selenocysteine or the selenomethionine spectra, or a combination of the two. These model spectra share a ‘white line’ peak energy of ~12 661 eV, consistent with the sample spectra, as well as a significantly higher energy peak at ~12 667 eV (second organo-Se peak). The selenoether amino acids are difficult to distinguish and cannot serve as perfect models for other related Se-containing amino acids, such as SeCT which has been implicated in *C. decipiens* previously (Peterson and Butler, 1971). Evidence for the presence of oxidized forms of selenium was found by modelling minor contributions of either dimethylselenoxide or selenite. Concentrations of Se in XAS-analysed plant organs are shown in Supplementary Data Table S11.

**Table 2.** Results of linear combination fitting of alternative model compound spectra to Se K-edge XANES spectra of fresh *C. decipiens* Se-spiked samples. DMSeO, dimethylselenoxide; MeSeCys, Se-methylselenocysteine; SeMet, selenomethionine; CysSSeCys, sulfoselenocysteine.

Sample	Proportion of component fitted					*N*_tot_	Residual (×10^−3^)
	DMSeO	MeSeCys	SeMet	Selenite pH5	CysSSeCys		
Young leaf	0.21	0.66	−	−	0.15	1.02	2.77
Young leaf	−	−	0.68	0.10	0.23	1.01	5.53
Old leaf	0.24	0.63	−	0.03	0.13	1.03	2.47
Old leaf	0.09	0.77	−	−	0.16	1.02	3.36
Stem	0.16	0.80	−	−	0.07	1.03	3.36
Stem	0.06	0.96	−	−	−	1.02	5.70
Root	0.08	0.82	−	−	0.12	1.02	3.90
Root	0.29	0.57	−	−	0.14	1.00	3.80
Root	0.25	0.63	−	−	0.14	1.02	4.00
Root	0.07	0.94	−	−	−	1.02	4.93
Seed	0.07	0.51	0.43	−	−	1.02	4.93

## DISCUSSION

### Coelospermum decipiens*: a selenium hyperaccumulator from non-seleniferous soils?*

*Coelospermum decipiens* in the Cape York Peninsula of Queensland (Australia) is perhaps the most extreme hyperaccumulator on account of its bioconcentration factor (Knott and McCray, 1959). It has been generally assumed that Se (hyper)accumulation is restricted to seleniferous soils, such as those in Colorado–Wyoming in the USA and the Richmond area of Australia. The existence of Se hyperaccumulation in plants growing on normal (i.e. not seleniferous) soils opened the possibility for the discovery of hitherto unknown Se hyperaccumulator plants in Australia. However, for the first time, *C. decipiens* was reported growing on seleniferous soils. These soils originating from the habitat of *C. decipiens* around the Cooktown and Hope Vale area are the first instances of seleniferous soils reported from the Cape York Peninsula. This plant was always assumed to hyperaccumulate on Se-deficient soils from historical reports of soil testing, but the discovery of Se in Hope Vale soils indicates several possibilities: (1) Se in soils on the Cape York Peninsula is stronger and more widespread than previously assumed, or (2) the inland Hope Vale populations may hold the key to the origins of Se hyperaccumulation in this species. It should be noted that while bioavailable Se is recorded as extremely low here, it matched the range of bioavailable Se found in the seleniferous areas from Richmond, Queensland (Harvey *et al.*, 2024). There is an apparent surface geology of ferricrete/ferruginous duricrust near the area of highest total soil Se, which would play a role in the lower levels of bioavailable Se (Huang *et al*., 2023). It should also be noted that samples were taken near roadsides so there may be a strong degree of environmental disturbance, which may influence the relationship between the underlying geology and soil matter. Future studies ought to focus on undisturbed woodland with known *C. decipiens* populations.

Selenium accumulation was notably absent from all other species in the Morindeae tribe. At this time, *C. decipiens* is the only known hyperaccumulator in the Rubiaceae family. Therefore, the presence of Se in the soils of inland Hope Vale could explain why this plant developed the capacity for Se hyperaccumulation and retained that capacity even when soils were broadly deficient, with differing uptake capacities for populations (Knott and McCray, 1959). Selenium in soils is often reported as a total concentration, which often does not align with the actual bioavailable or soluble Se and which makes correlating Se accumulation in hyperaccumulators and their natural soil concentrations difficult at a fine scale (Statwick and Sher, 2017). However, broader-scale associations are evident, where both Se hyperaccumulators that are restricted to the seleniferous soils (such as *N. amplexicaulis*) and more generalist species (such as *Cardamine violifolia*) accumulate more Se when exposed to higher Se (Yuan *et al.*, 2013; Harvey *et al.*, 2024). It is possible that *C. decipiens* may fall into the latter category, though the capacity for bioconcentration on deficient soils, as seen in the specimens from the Elim Beach site, is still notable.

### Selenium ‘hyperconcentration’ in the seeds, a distinctive trait

Whilst most hyperaccumulators tend to have very high Se concentrations in the seeds and flowers, there is usually a pattern of high Se in younger leaves too (Lima *et al*., 2018; Harvey *et al*., 2020). The concentrations seen in *C. decipiens* seeds are uniquely high, with values of up to 28 000 µg Se g^−1^ being some of the highest Se concentrations found in nature. Additionally, the stark difference between the concentrations found in the seeds compared with all other tissues (including seed coat and mesocarp and floral tissues) suggest a level of adaptation in an ecosystem sense – to protect and inject the seeds with concentrated Se while allowing any seed distributor to not be poisoned from the outer coating, as they would likely digest these tissues. The trend of higher Se accumulation in the seeds and other reproductive tissues is commonly observed across all studied hyperaccumulators, but in these cases there is generally a comparable accumulation in most shoot tissues. The Se distribution in *C. decipiens* differs from typical Se hyperaccumulators, such as *Astragalus* spp., but mimics several famous seleniferous tropical tree species. High concentrations of Se in the seeds above all other tissues are known in certain trees from the Lecythidaceae family: *Lecythis ollaria* and *L. minor* (coco de mono/monkeypot nuts), and *Bertholletia excelsa* (Brazil nut) (Mori, 1970; Chang *et al*., 1995; Hammel *et al*., 1996; Müller and Desel, 2010; Németh *et al*., 2013). Brazil nuts have long been advertised as a source of dietary Se, but experts in Se plants have cautioned against too high consumption of Lecythidaceae seeds, as exemplified by rare cases where *Lecythis* seed ingestion has resulted in human selenosis (Thomson *et al*., 2008; Müller and Desel, 2010). There are major differences in Se accumulation in these species though with and Brazil nuts tested from two different sampling locations in Brazil had significantly different Se concentrations (Chang *et al*., 1995); though compared with other Lecythidaceae Se accumulators *B. excelsa* typically has lower Se concentrations (Németh *et al*., 2013). *Bertholletia excelsa* and *Allantoma lineata* were found to have similarly lower concentrations of Se in their seeds, whereas *Lecythis pisonis* was capable of hyperaccumulation in the seeds (Andrade *et al*., 1999). *Lecythis ollaria* has been recorded with exceptional concentrations of Se in the seeds – up to 12 000 µg Se g^−1^ (Hammel *et al*., 1996). One source recorded >2 % Se in the dried defatted nuts of this species, though information on the number of seeds and preparation methods for the analysis was not available (Kerdel-Vegas, 1966). Consequently, *C. decipiens* could be placed as a comparable hyperaccumulator to *L. ollaria*, and both are often overlooked in the field of Se hyperaccumulator research.

### *Getting to grips with selenium (hyper)accumulation in* Coelospermum decipiens

Apart from the extreme concentrations of Se in the seeds, concentrations in leaf and stem tissues from field specimens were often above the concentrations expected from ‘normal’ plant species, particularly for the Hope Vale specimens (Terry *et al*., 2000; White, 2016). It should be noted that the strong presence of Se in the marginal leaf tissue could both be an indicator of herbivory defence and might be an adapation to limit toxicity in the photosynthetically active parts of the leaf tissues (Freeman *et al*., 2006*a*, 2010; Galeas *et al*., 2008; Quinn *et al*., 2008, 2010). The leaf this pattern was observed in had developed under conditions of elevated Se in the substrate, unlike its older leaf counterparts, which had already matured prior to dosing; consequently Se presence may more likely be attributed to developmental exposure instead of preferential sequestration in younger leaves. Selenium distribution in the leaf periphery has been observed in *C. violifolia* (Cui *et al*., 2018), and in marginal/apical leaf epidermal cell clusters in *Stanleya pinnata* (Freeman *et al*., 2006*b*, 2010). However, the distribution seen in *C. decipiens* leaf margins is not localized at the periphery or in clusters, as seen in these other two hyperaccumulators – the leaf margin appears to be a particular structure that sequesters all observable Se in the tissue. This is not the lateral loop vein common in the leaves of Rubiaceae species, which is observable in *C. decipiens* and is devoid of Se, though it could be a different type of vein, as suggested by the internal Se distribution. Alternatively, the Rubiaceae species *Coprosma obconica* has been observed with ‘thickened and recurved’ leaf margins, alongside a notably thick lateral loop vein (de Lange and Gardner, 2002), though this is not a feature observed in *C. decipiens*.

Comparatively, the natural collected specimens from the Weipa population had considerably lower Se in all tissues, i.e. below the historical hyperaccumulation threshold for Se recorded by Knott and McCray (1959), but were still elevated beyond the concentrations expected from ‘normal plants’. There are several possible explanations for this (if the plants were growing on Se-deficient soils, their uptake would be limited by their access to Se), such as in the historic investigations of a *C. decipiens* population 600 km north-north-west of Cooktown, which hosted hyperaccumulating specimens on low Se soils (Knott and McCray, 1959). It is possible that spatially distinct populations vary in their hyperaccumulating traits; this population effect is seen in other hyperaccumulating species, such as in *Noccaea* spp. (Assunção *et al*., 2003). Additionally, the previous survey also noted that selenosis in cattle occurred after they grazed on new foliage that resprouted after seasonal fires, so seasonality may play a strong role in Se concentration in the populations.

### *New insights into the biochemistry of selenium in* Coelospermum decipiens

A certain proportion of Se across all tissues was classified as organic Se (in this case fitted to SeMet and MeSeCys), which is present in higher proportions in Se accumulators compared with non-accumulators (Pilon-Smits *et al*., 1999; Pickering *et al*., 2003; Schiavon and Pilon-Smits, 2016). The XANES and XAFS (X-ray absorption fine structure) spectrum methodologies used in this research are not particularly effective at distinguishing between organo-Se compounds due to strong similarities between form spectra of organo-Se compounds, as identical methods used for *N. amplexicaulis* also did not distinguish between forms (M.-A. Harvey, unpubl. res.). Consequently, what is fitted here cannot be accurately quoted as the main form(s) of organo-Se in this plant without validation through methods such as LC–MS. Previous explorations into the biochemical forms of Se in *C. decipiens* noted the overwhelming presence of SeCT through extraction and electrophoresis methods (Peterson and Butler, 1971). Notably, the same researchers and techniques used to distinguish SeCT in this species were also able to distinguish a significant proportion of SeCT in *N. amplexicaulis* (Peterson and Butler, 1967). Recent investigations of *N. amplexicaulis* using LC–MS validated Peterson and Butler’s discovery of SeCT, suggesting an additional layer of validity to the assessment of SeCT in *C. decipiens* (M.-A. Harvey, unpubl. res.). It has previously been suggested that SeCT is a slightly more toxic form of Se than MeSeCys to accumulate; in this case, the high proportion of MeSeCys and accumulation capacity of *Astragalus* species were compared with the lower concentrations of Se in *C. decipiens* leaf tissues to support this hypothesis (Freeman *et al*., 2010). Comparatively, the seeds may tell a more complex story; it has been found that the protein-rich seeds of *B. excelsa* are concentrated in SeMet with some SeCys components as well, most of which were bound in proteins (Kannamkumarath *et al*., 2002; Dumont *et al*., 2006). Analysis of the Se metabolome of the seeds of *L. minor* revealed a varied collection of Se-amino acids (where derivatives of SeMet and selenohomocysteine/SeCT were most abundant) as well as polyselenide compounds (Dernovics *et al*., 2007; Németh *et al*., 2013). However, investigations into *L. ollaria* have confirmed a strong presence of SeCT in the seeds, along with inorganic forms, as may be the case for *C. decipiens* (Kerdel-Vegas *et al*., 1965; Ferri *et al*., 2004). Similar analysis of *C. decipiens* seeds would be needed to determine whether the chemical form of Se in the seeds is different from the leaves, and the diversity of Se metabolic pathways within this plant.

### *Future uses of* Coelospermum decipiens *by indigenous communities*

*Coelospermum decipiens*’ exceptional bioconcentration of Se into easily harvestable fruits may present some unique opportunities for a new local industry. Specifically, products derived from this species could be used to make Se-rich extracts for use in agricultural biofortification, as the Se compounds in Se hyperaccumulators have been shown to have low toxicity and very fast absorbance in food crops (Bañuelos *et al*., 2015). Alternatively, purified Se could be produced for use in pharmaceutical supplementation for Se deficiency. As *C. decipiens* is a plant with ethnobotanical significance, any venture involving (commercial) research and development activities with this plant requires meaningful partnership and benefit sharing with the local indigenous communities across the Cape York Peninsula, particularly the Guugu Yimithirr people of Hope Vale, who hold native title in the region and allowed us to conduct field research on their land. Additionally, further directions in research could integrate the other members of the Morindae tribe with unique ethnobotanical and pharmaceutical traits. While *C. decipiens* has been used as a dye and contraceptive medicine, its properties and uses may overlap with those of *Coelospermum reticulatum*, and other cultures have used tissues from *Morinda* species for dyes (Morton, 1992; Bhuyan and Saikia, 2003). Additionally, *Morinda reticulata* from India has traditionally been used medicinally, and extracts of the plant revealed a variety of phytochemicals with notable pharmacological properties, including effective antioxidants (Nair *et al*., 2012; Asirvatham and Usha, 2017; Nair and Padmaja, 2020). The current study into medicinal compounds in *M. reticulata* and the unique Se hyperaccumulation in *C. decipiens* present unique molecular and biochemical traits in this tribe that should be further investigated to understand the potential benefits of these plants.

## SUPPLEMENTARY DATA

Supplementary data are available at *Annals of Botany* online and consist of the following. Figure S1: examples of landscapes at different localities, inland localities at Endeavour Battlecamp Road (a) and Isabella McIvor Road (b), and the Elim beach locality (c) with coastal sandy forest. Figure S2: linear models showing the correlation between total soil Se from the root zone of *C. decipiens* (from ColdBlock analysis) and Se concentration in the aerial tissues of *C. decipiens* (log transformed). Figure S3: linear models showing the correlation between total soil Se from the root zone of *C. decipiens* (from ColdBlock analysis) and Se in the floral and fruit tissues of *C. decipiens* (log-transformed). Figure S4: *Coelospermum decipiens* from Weipa in North Queensland. (A) Whole plant. (B) Root crown. (C) Root cross section. (D) Seed pods with enlarged white calycophylls. Figure S5: dosing trial of *C. decipiens*. (A) Cutting during trial. (B) Newly grown root at harvest. (C) ICP–AES samples for analysis. Red samples are acid digests of roots specifically. Table S1: Se concentrations in Morindeae tribe species from Queensland Herbarium. Table S2: total bulk elements from field soil samples from Hope Vale and Cooktown (in milligrams per kilogram). Table S3: bulk major elemental concentrations in field-collected plant tissues in *C. decipiens* (values are given as means and ranges in micrograms per gram, and *n* is the number of samples). Table S4: bulk minor elemental concentrations in field-collected plant tissues in *C. decipiens* (values are given as ranges and means in micrograms per gram, and *n* is the number of samples). Table S5: bulk elemental concentrations in herbarium specimen plant tissues of *C. decipiens* (in micrograms per gram). Samples taken from AQ325702. Table S6: bulk major elemental concentrations in field-collected plant tissues in *C. decipiens* (values are given in means and ranges in micrograms per gram, and *n* is the number of samples). Table S7: bulk minor elemental concentrations in field-collected plant tissues in *C. decipiens* (values are given as means and ranges in micrograms per gram, and *n* is the number of samples). Table S8: bulk major elemental concentrations in glasshouse-grown tissues in *C. decipiens* (values are given as means and ranges in micrograms per gram, and *n* is the number of samples). Table S9: bulk minor elemental concentrations in glasshouse-grown tissues in *C. decipiens* (values are given as means and ranges in micrograms per gram, and *n* is the number of samples). Table S10: output of Kruskal–Wallis, ANOVA and Scheirer–Ray–Hare tests and Wilcoxon *post hoc* test for the dosing trial samples. Table S11: bulk elemental concentrations in *C. decipiens* tissues analysed using XAS (values are given as means and ranges in micrograms per gram, and *n* is the number of samples).

mcae103_suppl_Supplementary_Material
