# Supplementary material for: The curious case of selenium hyperaccumulation in Coelospermum decipiens from the Cape York Peninsula (Queensland, Australia)
Source: Ann Bot. 2024 Jun 25;134(5):769–86. doi: 10.1093/aob/mcae103 (PMC11560376; doi:10.1093/aob/mcae103)
Supplement: mcae103_suppl_Supplementary_Material [file mcae103_suppl_supplementary_material.doc]

**SUPPLEMENTARY INFORMATION**

**The curious case of selenium hyperaccumulation in *Coelospermum decipiens* from the Cape York Peninsula (Queensland, Australia)**

Maggie-Anne Harvey^1,2^, Katherine Pinto Irish^2^, Hugh H. Harris^3^, Peter D. Erskine^2^,

Antony van der Ent^1,2,4*^

^1^Laboratory of Genetics, Wageningen University and Research, The Netherlands.

^2^Centre for Mined Land Rehabilitation, Sustainable Minerals Institute,

The University of Queensland, Australia.

^3^Department of Chemistry, The University of Adelaide, Australia.

^4^Université de Lorraine, INRAE, LSE, F-54000 Nancy, France.

*Corresponding author: A. van der Ent (antony.vanderent@wur.nl)

**
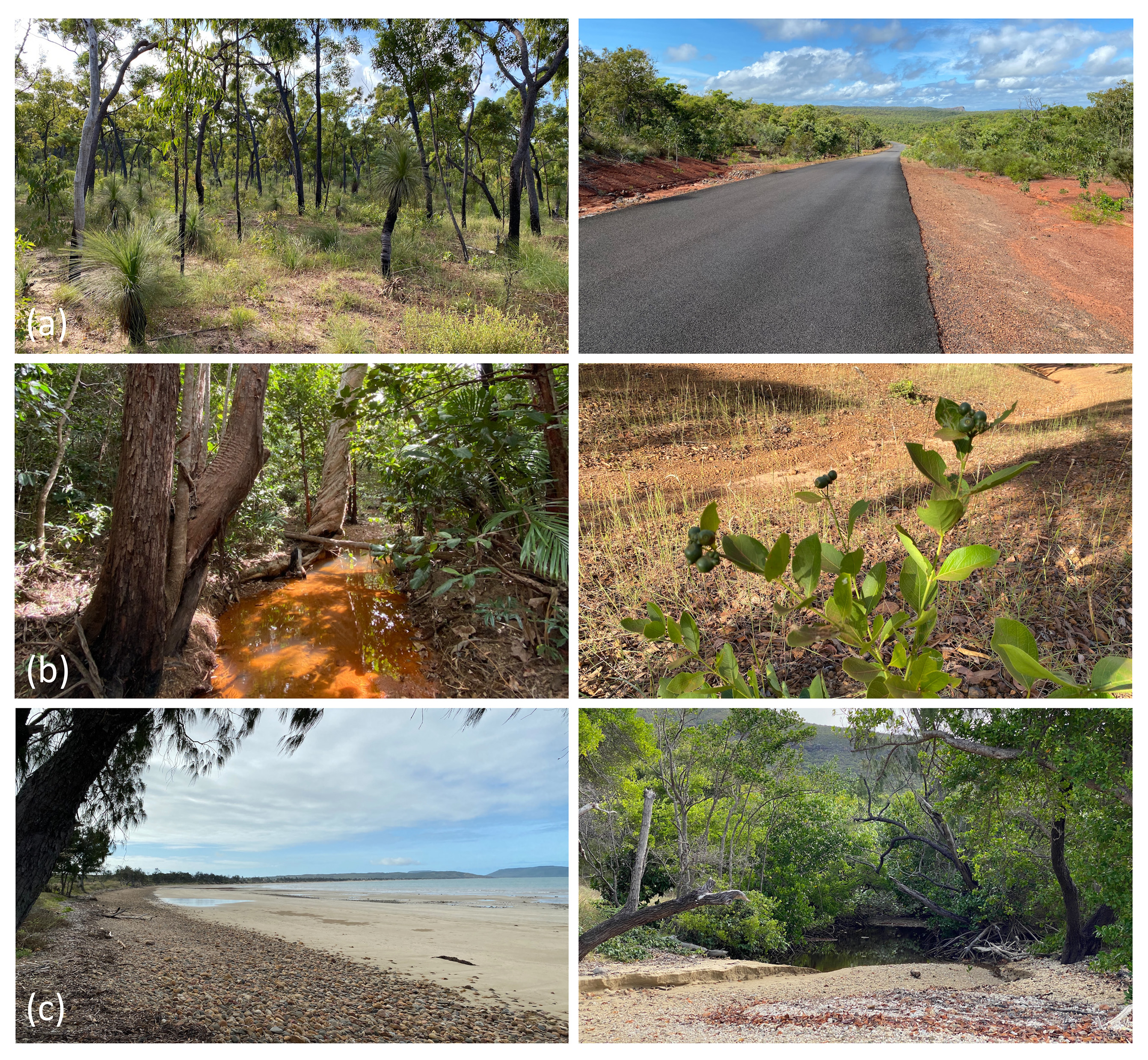
**

**Supplementary Information Figure 1**. Examples of landscapes at different localities, inland localities at Endeavour Battlecamp Road (a) and Isabella McIvor Road (b), and the Elim beach locality (c) with coastal sandy forest.


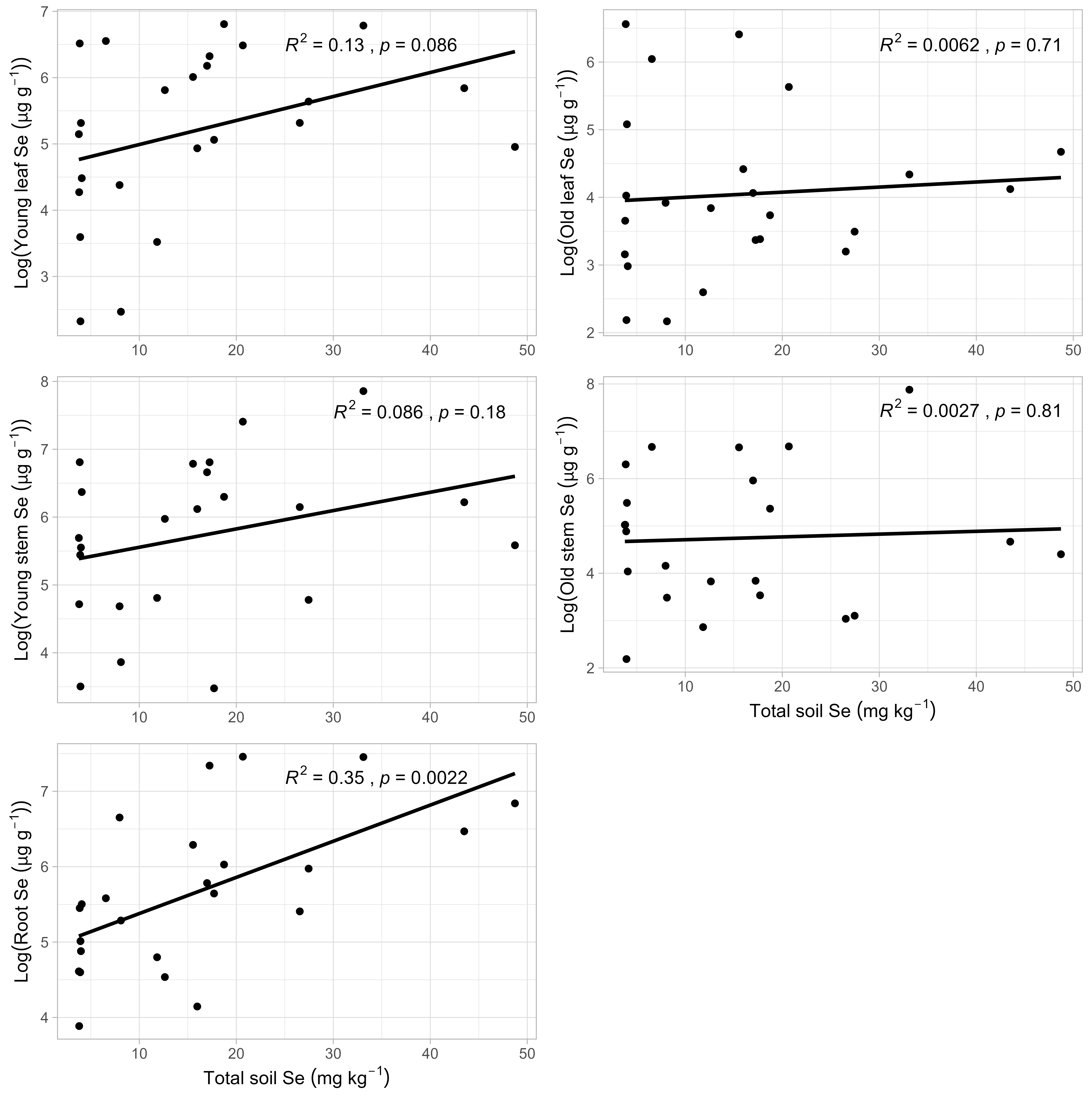
**Supplementary Information Figure 2.** Linear models showing the correlation between total soil Se from the rootzone of *C. decipiens* (from ColdBlock analysis) and Se concentration in the aerial tissues of *C. decipiens* (log transformed). R^2^ and *p*-value displayed on graph.


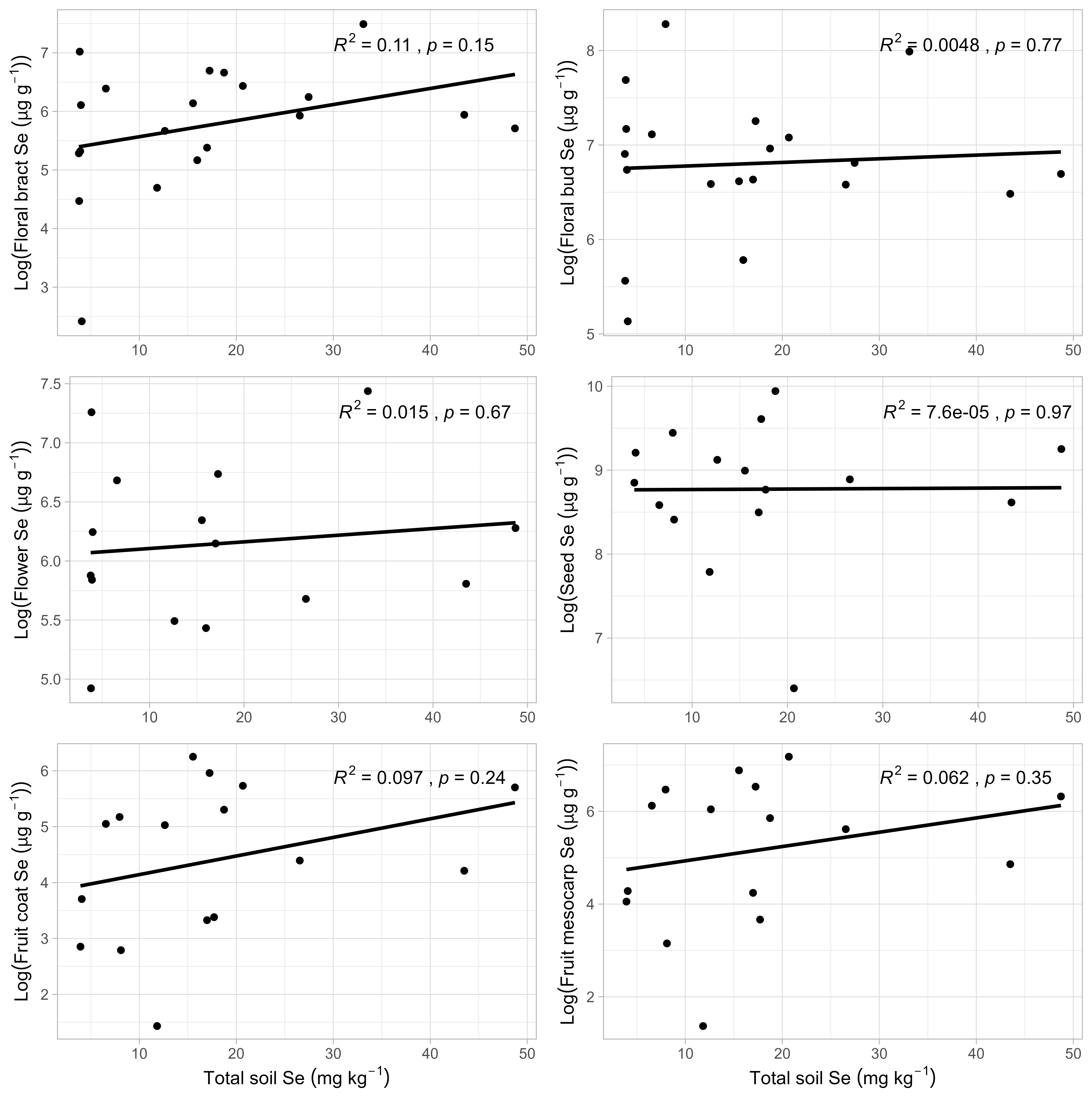


**Supplementary Information Figure 3.** Linear models showing the correlation between total soil Se from the rootzone of *C. decipiens* (from ColdBlock analysis) and Se in the floral and fruit tissues of *C. decipiens* (log transformed). R^2^ and *p*-value displayed on graph


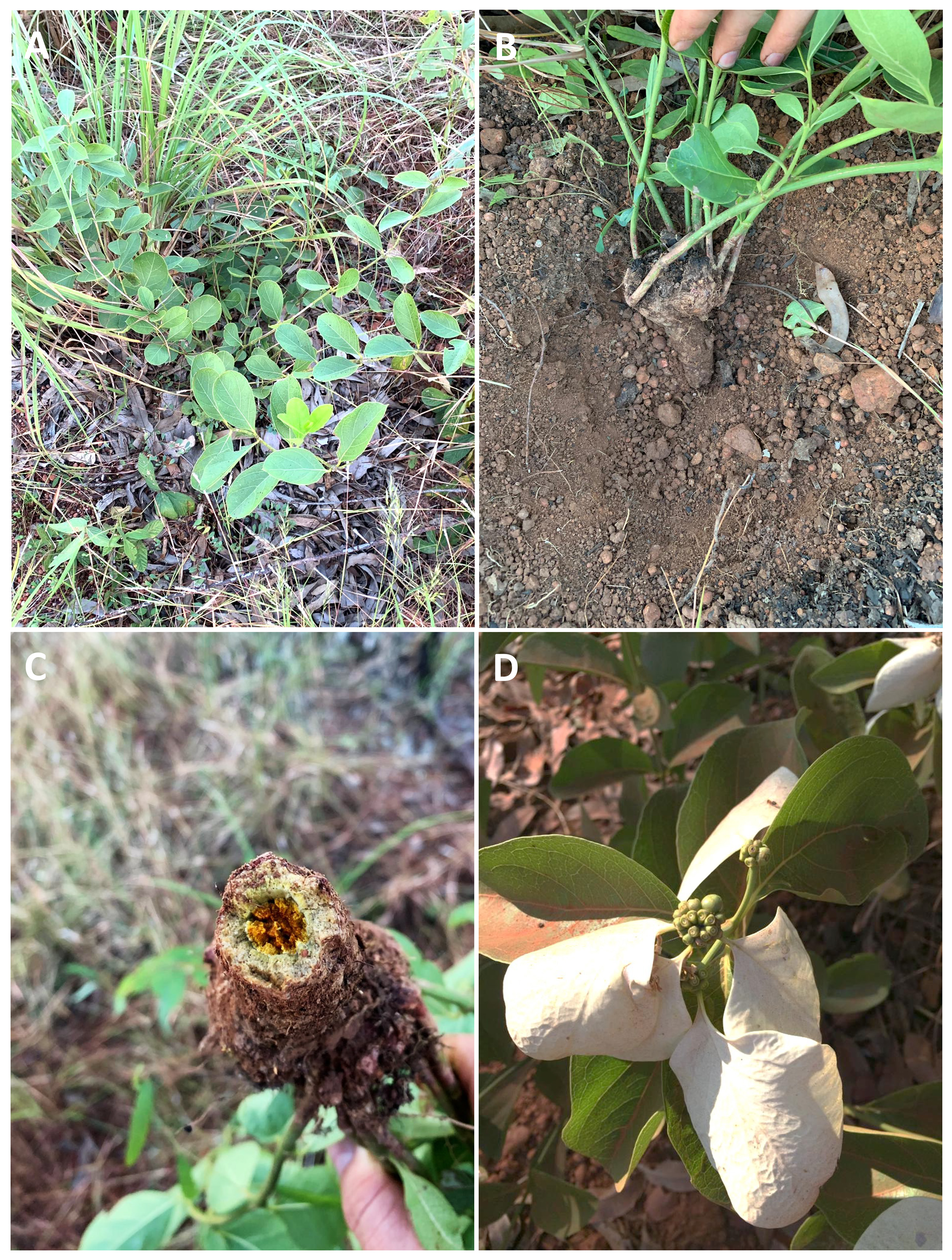


**Supplementary Information Figure 4.** *Coelospermum decipiens* from Weipa in North Queensland. (A) Whole plant, (B) Root crown. (C) Root cross section. (D) Seedpods with enlarged white calycophylls.

**
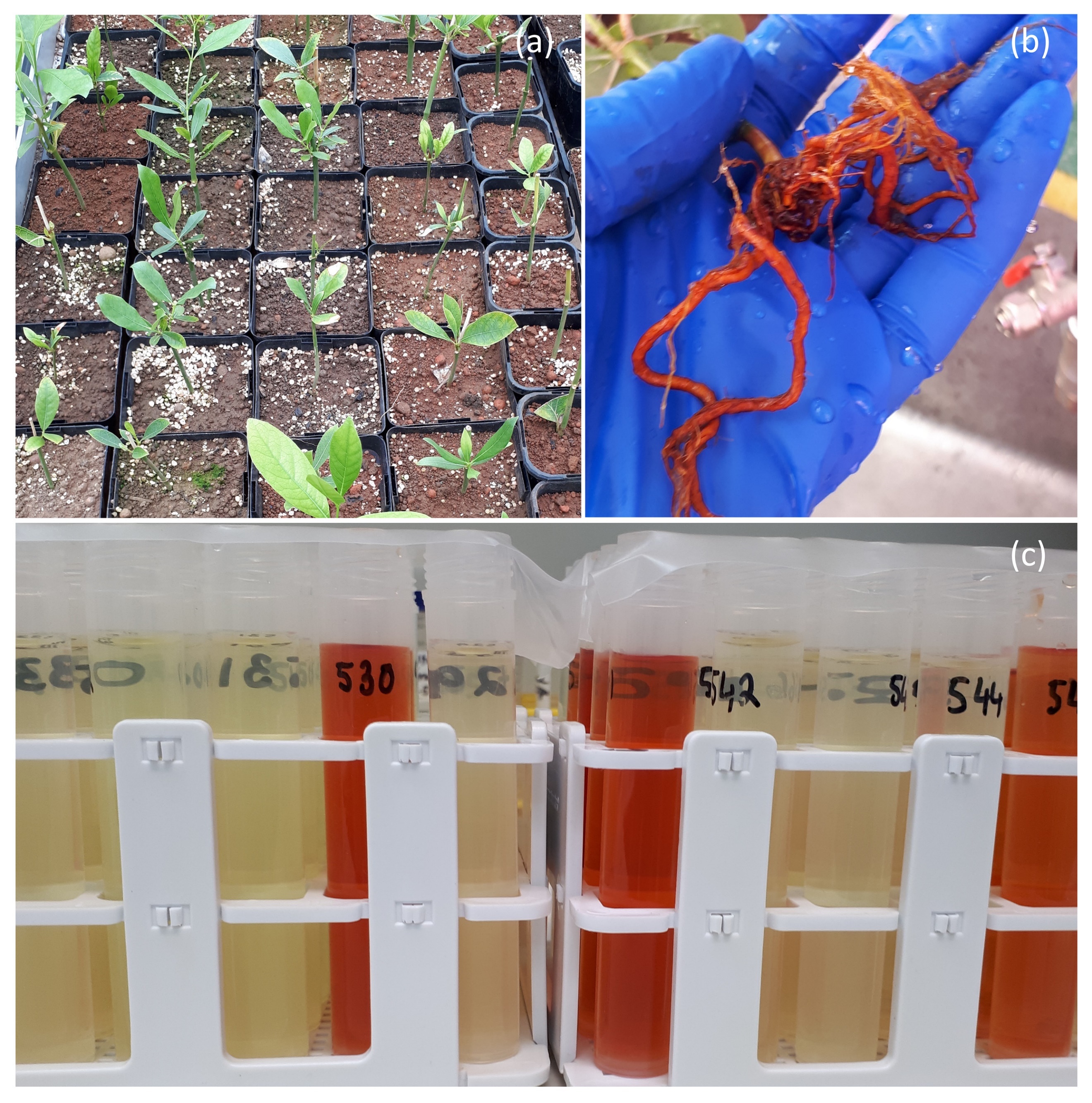
**

**Supplementary Information Figure 5**. Dosing trial of *Coelospermum decipiens*. (a) cutting during trial (b) newly grown root at harvest (c) ICP-AES samples for analysis–red samples are acid digests of roots specifically.

**Supplementary Information Table 1.** Se concentrations in Morindeae tribe species from Queensland Herbarium. Selenium presented in µg g^-1^ for range and average. *n*= number of specimens. *P* = p -value of difference in Se levels between *C. decipiens* and Morindeae species from Tukey’s HSD.

| **Species** | **n** | **Range** | **Average** | ***p*** |
| --- | --- | --- | --- | --- |
| *Coelospermum dasylobum* | 29 | 0­–7 | 1 | 0.00 |
| *Coelospermum decipiens* | 223 | 0­–639 | 72 | 1.00 |
| *Coelospermum paniculatum* | 118 | 0­–7 | 1 | 0.00 |
| *Coelospermum purpureum* | 69 | 0­–7 | 2 | 0.00 |
| *Coelospermum reticulatum* | 364 | 0­–10 | 1 | 0.00 |
| *Gynochthodes ammitia* | 58 | 0­–7 | 1 | 0.00 |
| *Gynochthodes australiensis* | 2 | 0­–0 | 0 | 0.32 |
| *Gynochthodes canthoides* | 110 | 0­–7 | 1 | 0.00 |
| *Gynochthodes constipata* | 14 | 0­–6 | 2 | 0.00 |
| *Gynochthodes jasminoides* | 257 | 0­–11 | 2 | 0.00 |
| *Gynochthodes oresbia* | 38 | 0­–8 | 3 | 0.00 |
| *Gynochthodes podistra* | 28 | 0­–8 | 2 | 0.00 |
| *Gynochthodes retropila* | 49 | 0­–8 | 3 | 0.00 |
| *Gynochthodes sessilis* | 30 | 0­–7 | 2 | 0.00 |
| *Gynochthodes umbellata* | 97 | 0­–7 | 1 | 0.00 |
| *Morinda bracteata* | 47 | 0­–9 | 2 | 0.00 |
| *Morinda citrifolia* | 143 | 0­–15 | 2 | 0.00 |

**Supplementary Information Table 2.** Total bulk elements from field soil samples from Hope Vale and Cooktown (in mg kg^-1^). Name is link with specific *C. decipiens* collection. Limit of detection (LOD) in mg kg^-1^ in the first row calculated from internal standards.

| **NAME** | **LOCALITY** | **Zn** | **Ca** | **Mn** | **Fe** | **K** | **P** | **S** | **Mg** | **V** | **Se** |
| --- | --- | --- | --- | --- | --- | --- | --- | --- | --- | --- | --- |
| **LOD** | **-** | 0.949 | 15.5 | 59.9 | 81.0 | 3.65 | 48.9 | 8.60 | 69.1 | 0.282 | 5.57 |
| coelo-2 | Endeavour Battlecamp | 3.91 | 106 | <LOD | 14650 | 21.0 | <LOD | 23.6 | <LOD | 20.3 | 4.07 |
| coelo-3 | Endeavour Battlecamp | 3.15 | 141 | <LOD | 11544 | 48.7 | <LOD | 40.4 | <LOD | 21.9 | 7.96 |
| coelo-4 | Endeavour Battlecamp | 4.29 | 230 | <LOD | 20949 | 126 | <LOD | 95.1 | 115 | 27.7 | 8.10 |
| coelo-5 | Endeavour Battlecamp | 4.28 | 184 | <LOD | 8375 | 42.8 | <LOD | 41.2 | <LOD | 11.9 | <LOD |
| coelo-6 | Endeavour Battlecamp | 3.54 | 236 | <LOD | 34644 | 114 | 73.1 | 83.9 | 120 | 42.0 | 17.7 |
| coelo-7 | Endeavour Battlecamp | 4.82 | 329 | <LOD | 34135 | 46.2 | 54.2 | 65.0 | 97.3 | 35.9 | 17.0 |
| coelo-8 | Endeavour Battlecamp | 17.7 | 833 | 537 | 48591 | 134 | 206 | 190 | 326 | 86.1 | 33.1 |
| coelo-9 | Endeavour Battlecamp | 8.70 | 291 | 350 | 31916 | 82.0 | 120 | 180 | 135 | 56.7 | 17.2 |
| coelo-10 | Endeavour Battlecamp | 13.6 | 749 | 246 | 38205 | 221 | 150 | 144 | 223 | 66.1 | 20.7 |
| coelo-11 | Endeavour Battlecamp | 6.03 | 227 | <LOD | 48700 | 111 | 88.5 | 167 | <LOD | 69.6 | 27.5 |
| coelo-12 | Isabella McIvor | 3.73 | 106 | <LOD | 13393 | 11.7 | <LOD | 52.6 | <LOD | 22.9 | 6.55 |
| coelo-13 | Isabella McIvor | 12.2 | 300 | 56.8 | 47157 | 132 | 74.7 | 75.8 | 394 | 77.2 | 26.5 |
| coelo-14 | Isabella McIvor | 12.8 | 156 | <LOD | 21666 | 164 | <LOD | 109 | 368 | 10.4 | 11.8 |
| coelo-15 | Isabella McIvor | 35.9 | 181 | 159 | 34348 | 215 | 118 | 14.5 | 1671 | 19.3 | 18.7 |
| coelo-16 | Isabella McIvor | 8.28 | 463 | 203 | 32633 | 220 | 89.2 | 125 | 265 | 35.5 | 15.5 |
| coelo-17 | Isabella McIvor | 4.74 | 106 | <LOD | 6182 | 26.0 | <LOD | 58.2 | <LOD | 8.18 | <LOD |
| coelo-18 | Isabella McIvor | 30.0 | 367 | 110 | 88748 | 64.4 | 185 | 180 | 431 | 116 | 48.7 |
| coelo-19 | Isabella McIvor | 31.9 | 396 | 152 | 86914 | 85.2 | 261 | 157 | 427 | 117 | 43.5 |
| coelo-20 | Isabella McIvor | 7.03 | 189 | 76.4 | 22241 | 70.3 | 63.2 | 49.3 | 105 | 35.7 | 12.6 |
| coelo-21 | Elim Beach | 4.23 | 201 | <LOD | 2256 | 73.1 | <LOD | 96.0 | 128 | 4.47 | <LOD |
| coelo-22 | Elim Beach | <LOD | 84.0 | <LOD | 995 | 27.1 | <LOD | 34.2 | <LOD | 2.48 | <LOD |
| coelo-23 | Elim Beach | 2.15 | 116 | <LOD | 3132 | 88.4 | <LOD | 26.7 | 76.8 | 5.38 | <LOD |
| coelo-24 | Elim Beach | 37.2 | 2503 | 460 | 24051 | 726 | 888 | 79.9 | 2968 | 31.7 | 16.0 |
| coelo-25 | Elim Beach | 11.9 | 287 | <LOD | 6931 | 372 | 101 | 84.9 | 618 | 10.4 | <LOD |

**Supplementary Information Table 3.** Bulk major elemental concentrations in field-collected plant tissues in *Coelospermum decipiens* (values are given in means and ranges in µg g^-1^, and *n* is the number of samples). Samples collected from Hope Vale, Far North Queensland. The digests were analysed with Inductively Coupled Plasma Atomic Emission Spectroscopy (ICP-AES). Limit of detection (LOD) in µg g^-1^ calculated from internal standards. Means calculated using LOD/√2 to replace values <LOD.

| **TISSUE** | ***n*** | **Na** | **Mg** | **K** | **Ca** | **P** | **S** |
| --- | --- | --- | --- | --- | --- | --- | --- |
| **LOD** | ***-*** | 118 | 21.0 | 6.36 | 3.39 | 16.1 | 62.2 |
| Young Leaf | 24 | <LOD–733  267 | 2257–6174  3635 | 7030–25097  15589 | 1694–13168  5653 | 307–1373  769 | 1067–3123  1864 |
| Old Leaf | 24 | <LOD–756  367 | 1966–6798  3537 | 5666–19517  12133 | 2855–13707  6174 | 287–788  501 | 986–4447  1825 |
| Young Stem | 24 | <LOD–681  272 | 940–4144  2125 | 9078–25358  17187 | 1516–8426  3308 | 246–1290  656 | 680–2721  1486 |
| Old Stem | 24 | <LOD–586  210 | 370–3721  1538 | 6871–18437  10715 | 729–3113  1748 | 189–660  334 | 347–1601  803 |
| Roots | 24 | <LOD–843  312 | 419–3606  1350 | 4332–12702  9071 | 1637 -14888  7587 | 229–1141  458 | 569–1463  976 |
| Flowers | 15 | <LOD–879  434 | 1234–2749  1803 | 15230–27879  21001 | 2565–6926  3921 | 1012–2256  1537 | 1535–2435  1868 |
| Flower buds | 20 | <LOD–1052  196 | 3214–13056  5865 | 13634–39908  23944 | 2088–16115  8601 | 759–2648  2007 | 2549–4605  3626 |
| Floral bract | 20 | <LOD–2301  416 | 1438–4045  2535 | 11154–30985  18326 | 1262–8293  4472 | 231–1504  997 | 917–2727  1554 |
| Seed | 16 | <LOD–109  376 | 2093–4270  2899 | 2471–17460  5778 | 731–18479  3065 | 2757–6103  4514 | <LOD–3908  2577 |
| Mesocarp | 16 | <LOD | 880–2558  1726 | 12451–25009  18178 | 816–7971  2302 | 198–1642  727 | 604–1725  1205 |
| Seed coat | 16 | <LOD–229  95.6 | 348–2526  912 | 6981–12443  10213 | 318–6550  1380 | 142–1138  414 | 326–1493  582 |

**Supplementary Information Table 4.** Bulk minor elemental concentrations in field-collected plant tissues in *Coelospermum decipiens* (values are given in ranges and means in µg g^-1^, and *n* is the number of samples). Samples collected from Hope Vale, Far North Queensland. The digests were analysed with Inductively Coupled Plasma Atomic Emission Spectroscopy (ICP-AES). The average limit of detection (LOD), in µg g^-1^, was calculated from internal standards. LOD for Se calculated from axial mode. Means calculated using LOD/√2 to replace values <LOD.

| **TISSUE** | ***N*** | **Fe** | **Mn** | **Cu** | **Zn** | **Se** |
| --- | --- | --- | --- | --- | --- | --- |
| **LOD** | ***-*** | 7.4 | 7.87 | 1.95 | 2.09 | 0.65 |
| Young Leaf | 24 | 29.2–343  97.0 | 116–280  177 | <LOD–11.1  5.14 | 4.87–16.5  11.2 | <LOD–905  316 |
| Old Leaf | 24 | 43.1–602  157 | 106–392  227 | <LOD–6.73  3.28 | 4.60–17.7  9.62 | <LOD–707  124 |
| Young Stem | 24 | 15.7–180  60.2 | 87.1–481  179 | <LOD–15.4  6.93 | 3.17–31.9  13.7 | 32–2584  534 |
| Old Stem | 24 | 12.1–122  47.6 | 47.9–1143  245 | <LOD–8.28  3.79 | 2.68–20.4  9.32 | <LOD–2638  320 |
| Roots | 24 | 64.5–8220  808 | 40.6–1071  284 | <LOD–8.53  3.77 | 3.12–26.0  9.92 | 49–1735  470 |
| Flowers | 15 | 37.8–4494  731 | <LOD–149  83.9 | <LOD–25.0  11.8 | 15.2–39.1  24.3 | 137–1698  585 |
| Flower buds | 20 | 33.3–4467  730 | 147–519  277 | 2.63–26.1  13.0 | 17.6–48.2  27.1 | 170– 3942  1159 |
| Floral bract | 20 | 30.3–1017  387 | 67.1–190  125 | <LOD–21.1  8.57 | 5.74–22.2  13.1 | <LOD–1789  475 |
| Seed | 16 | <LOD–294  63.5 | <LOD–190  144 | <LOD–50.1  26.7 | <LOD–56.2  43.5 | 602–20 777  8816 |
| Mesocarp | 16 | 31.4–133  50.3 | 33.9–132  70.5 | <LOD–19.1  5.52 | 3.90–16.2  9.24 | <LOD–1310  379 |
| Seed coat | 16 | 13.4–182  32.7 | 12.4–91.0  36.6 | <LOD–14.1  3.93 | 2.94–13.4  6.10 | <LOD–518  155 |

**Supplementary Information Table 5.** Bulk elemental concentrations in herbarium specimen plant tissues of *Coelospermum decipiens* (in µg g^-1^)*.* Samples taken from AQ 325702. The digests were analysed with Inductively Coupled Plasma Atomic Emission Spectroscopy (ICP-AES). Limit of detection (LOD) in µg g^-1^ calculated from internal standards.

| **Tissue** | **Na** | **Mg** | **K** | **Ca** | **P** | **S** | **Fe** | **Mn** | **Cu** | **Zn** | **Se** |
| --- | --- | --- | --- | --- | --- | --- | --- | --- | --- | --- | --- |
| **LOD** | 79 | 0 | 2 | 4 | 26 | 599 | 2 | 4 | 4 | 0 | 6 |
| Flower | 226 | 7188 | 33736 | 9585 | 4005 | 5221 | 46 | 252 | 6 | 44 | 3343 |
| Old Leaf | 156 | 4566 | 30145 | 5210 | 1898 | 1981 | 56 | 136 | 4 | 19 | 1463 |
| Seedcoat | 80 | 1907 | 14988 | 2477 | 1074 | 1408 | 23 | 71 | 4 | 4 | 350 |
| Seeds | 101 | 826 | 12528 | 2623 | 777 | 901 | 15 | 33 | 4 | 3 | 260 |

**Supplementary Information Table 6.** Bulk major elemental concentrations in field-collected plant tissues in *Coelospermum decipiens* (values are given in means and ranges in µg g^-1^, and *n* is the number of samples). Samples collected from Weipa, Far North Queensland. The digests were analysed with Inductively Coupled Plasma Atomic Emission Spectroscopy (ICP-AES). Limit of detection (LOD) in µg g^-1^ calculated from internal standards. Means calculated using LOD/√2 to replace values <LOD.

| **Tissue** | ***n*** | **Na** | **Mg** | **K** | **Ca** | **P** | **S** |
| --- | --- | --- | --- | --- | --- | --- | --- |
| **LOD** | - | 79 | 0 | 2 | 4 | 26 | 599 |
| Young Leaf | 7 | 175  (<LOD–582) | 5408  (3244–7852) | 6052  (3259–13821) | 9185  (8046–10388) | 695  (363–1684) | 1918  (1072–3449) |
| Old Leaf | 7 | 271  (<LOD–647) | 5374  (3420–10884) | 4493  (2875–7787) | 9962  (5378–14652) | 527  (404–641) | 1511  (1102–1771) |
| Stems | 7 | 276  (139–492) | 3046  (1945–4563) | 6493  (4826–9030) | 5629  (3409–11710) | 392  (259–547) | 1581  (1209–2500) |
| Roots | 7 | 522  (215–881) | 3155  (1563–5829) | 3995  (2250–5931) | 15746  (8456–24602) | 307  (26–425) | 1385  (812–2065) |
| Seedcoat | 5 | 61  (<LOD–87) | 2445  (1334–3218) | 10818  (7997–16095) | 2835  (1593–4449) | 587  (318–778) | 1139  (848–1689) |
| Seeds | 5 | <LOD | 1509  (1096–2380) | 8547  (5934–12144) | 3017  (2587–3962) | 978  (563–1979) | 961  (423–1720) |

**Supplementary Information Table 7.** Bulk minor elemental concentrations in field-collected plant tissues in *Coelospermum decipiens* (in µg g^-1^, values are given in means and ranges, and *n* is the number of samples). Samples collected from Weipa, Far North Queensland. The digests were analysed with Inductively Coupled Plasma Atomic Emission Spectroscopy (ICP-AES). Limit of detection (LOD) in µg g^-1^ calculated from internal standards. Means calculated using LOD/√2 to replace values <LOD.

| **Tissue** | ***n*** | **Fe** | **Mn** | **Cu** | **Zn** | **Se** |
| --- | --- | --- | --- | --- | --- | --- |
| **LOD** | - | 2 | 4 | 4 | 0 | 6 |
| Young Leaf | 7 | 78  (44–142) | 184  (129–266) | 3  (2–4) | 8  (5–11) | 25  (9–80) |
| Old Leaf | 7 | 191  (61–325) | 206  (158–335) | 3  (2–4) | 6  (3–9) | 11  (<LOD–21) |
| Stems | 7 | 151  (16–294) | 233  (108–478) | 3  (2–4) | 4  (3–7) | 17  (7–31) |
| Roots | 7 | 758  (247–2685) | 152  (109–232) | 3  (2–6) | 4  (3–8) | 108  (59–260) |
| Seedcoat | 5 | 44  (19–84) | 89  (47–126) | 3  (2–3) | 8  (5–9) | 68  (<LOD–270) |
| Seeds | 5 | 19  (13–28) | 58  (42–92) | 3  (1–6) | 11  (8–11) | 320  (9–565) |

**Supplementary Information Table 8**. Bulk major elemental concentrations in glasshouse grown tissues in *Coelospermum decipiens* (values are

| Tissue | Se | **Na** | **Mg** | **K** | **Ca** | **P** | **S** |
| --- | --- | --- | --- | --- | --- | --- | --- |
| **LOD** |  | 79 | 0 | 2 | 4 | 26 | 599 |
| Young Leaves | 0 | 725  (<LOD–3027) | 5443  (4013–8218) | 7580  (3047–16478) | 11434  (5480–18114) | 428  (<LOD–1600) | 1696  (1046–3763) |
| Old leaves | 0 | 1233  (284–5595) | 5550  (3192–7946) | 7095  (2299–22464) | 12118  (5659–18541) | 375  (<LOD–1002) | 1303  (778–2178) |
| Stem | 0 | 1557  (888–3783) | 4112  (1524–7971) | 4853  (2116–9781) | 3500  (1548–5936) | 202  (<LOD–823) | 863  (<LOD–1284) |
| Root | 0 | 909  (498–1700) | 3413  (1927–4700) | 9464  (6196–17080) | 8114  (4019–13061) | 423  (140–945) | 991  (<LOD–1532) |
| Young Leaves | 5 | 1186  (<LOD–3223) | 6444  (4158–8596) | 7762  (1455–27825) | 12028  (4987–17512) | 715  (<LOD–3224) | 1690  (778–5116) |
| Old leaves | 5 | 1531  (393–2298) | 6762  (4698­-8155) | 3516  (2046–6019) | 13349  (6843–18427) | 398  (203–606) | 1181  (628–1713) |
| Stem | 5 | 2125  (275–4612) | 4951  (2800–8198) | 3494  (1558–5587) | 3921  (2150–6021) | 282  (169–485) | 849  (<LOD–1565) |
| Root | 5 | 848  (383–1564) | 3403  (2445–6082) | 10505  (6261–17969) | 7023  (4296–10483) | 590  (208–1363) | 1072  (<LOD–1817) |

given in means and ranges in µg g^-1^ and *n* is the number of samples). Samples derived from field-collected cuttings grown in Weipa soil, watered twice weekly with Se (in µg g^-1^) solution in the form of SeO_4_. The digests were analysed with Inductively Coupled Plasma Atomic Emission Spectroscopy (ICP-AES). Limit of detection (LOD) in µg g^-1^ calculated from internal standards. Means calculated using LOD/√2 to replace values <LOD in raw mg/L.

**Supplementary Information Table 9**. Bulk minor elemental concentrations in glasshouse grown tissues in *Coelospermum decipiens* (values are given in means and ranges in µg g^-1^, and *n* is the number of samples). Samples derived from field-collected cuttings grown in Weipa soil, watered twice weekly with Se (in µg g^-1^) solution in the form of SeO_4_ (Dose). The digests were analysed with Inductively Coupled Plasma Atomic Emission Spectroscopy (ICP-AES). Limit of detection (LOD) in µg g^-1^ calculated from internal standards. Means calculated using LOD/√2 to replace values <LOD in raw mg/L.

| **Tissue** | **Dose** | **Fe** | **Mn** | **Cu** | **Zn** | **Se** |
| --- | --- | --- | --- | --- | --- | --- |
| **LOD** |  | 2 | 4 | 1 | 0 | 6 |
| Young Leaves | 0 | 41  (18–151) | 162  (124–216) | 2  (<LOD–13) | 10  (3–21) | 41  (<LOD–358) |
| Old leaves | 0 | 52  (18–126) | 178  (124–243) | 2  (<LOD–5) | 10  (5–20) | 31  (<LOD–170) |
| Stem | 0 | 50  (23–90) | 166  (50–252) | 2  (<LOD–4) | 11  (4–32) | 10  (<LOD-22) |
| Root | 0 | 399  (183–793) | 128  (80–185) | 4  (1–8) | 18  (10–49) | 156  (19–427) |
| Young Leaves | 5 | 54  (25–142) | 198  (118–276) | 4  (<LOD–26) | 9  (4–36) | 124  (<LOD­–669) |
| Old leaves | 5 | 62  (22–111) | 208  (154–272) | 2  (<LOD–4) | 5  (2–9) | 24  (<LOD­–94) |
| Stem | 5 | 75  (43–124) | 197  (93–369) | 3  (<LOD–16) | 9  (2–44) | 15  (<LOD–38) |
| Root | 5 | 274  (159–450) | 121  (85–184) | 4  (2–8) | 12  (4–25) | 291  (69­–759) |

**Supplementary Table 10.** Output of Kruskal Wallis, ANOVA and Scheirer-Ray-Hare tests, and Wilcoxon *post-hoc* test for the dosing trial samples. Tests run in RStudio using rstatix and rcompanion. Tissue is the young leaf, old leaf, stem or root treatment is control or dosed.

| **Kruskal-Wallis** | **Df** | **N** | **Statistic** | **P value** | **Wilcoxon’s Test** | **n1 – n2** | **Statistic** | **P adjusted** |
| --- | --- | --- | --- | --- | --- | --- | --- | --- |
| Se~Treatment | 1 | 103 | 2.81 | 0.0935 | Young leaf – old leaf | 26 – 21 | 284 | 1 |
| Se~Tissue | 3 | 103 | 45.0 | 9.44e-10*** | Young leaf – stem | 26 – 28 | 392 | 1 |
|  |  |  |  |  | Young leaf – root | 26 – 28 | 112 | 0.000025 |
|  |  |  |  |  | Old leaf – stem | 21 – 28 | 326 | 1 |
|  |  |  |  |  | Old leaf – root | 21 – 28 | 30 | 0.00000000876 |
|  |  |  |  |  | Stem - root | 28 – 28 | 12 | 0.00000000301 |
| **ANOVA** | **Df** | **Sum Sq** | **F-val** | **P (>F)** |  |  |  |  |
| Tissue | 3 | 751074 | 20.253 | 3.14e-10 *** |  |  |  |  |
| Treatment | 1 | 85228 | 6.895 | 0.0101 * |  |  |  |  |
| Tissue:Treatment | 3 | 86472 | 2.332 | 0.0791 |  |  |  |  |
| Residuals | 95 | 1174359 | - | - |  |  |  |  |
| **Scheirer-Ray-Hare** | **Df** | **Sum Sq** | **H** | **P value** |  |  |  |  |
| Tissue | 3 | 39659 | 44.428 | 0.00000*** |  |  |  |  |
| Treatment | 1 | 2038 | 2.283 | 0.13077 |  |  |  |  |
| Tissue:Treatment | 3 | 557 | 0.624 | 0.89083 |  |  |  |  |
| Residuals | 95 | 48323 | - | - |  |  |  |  |

n is number of samples.

**Supplementary Information Table 11**. Bulk elemental concentrations in *Coelospermum decipiens* tissues analysed using XAS (values are given in means and ranges in µg g^-1^, and *n* is the number of samples). Samples derived from field-collected cuttings grown in Weipa soil, watered twice weekly with Se (in µg g^-1^) solution in the form of SeO_4_. Seed and seedcoat were field specimens from Weipa. The digests were analysed with Inductively Coupled Plasma Atomic Emission Spectroscopy (ICP-AES). Limit of detection (LOD) in µg g^-1^ calculated from internal standards.

| **TISSUE** | ***n*** | **Na** | **Mg** | **K** | **Ca** | **P** | **S** | **Fe** | **Mn** | **Cu** | **Zn** | **Se** |
| --- | --- | --- | --- | --- | --- | --- | --- | --- | --- | --- | --- | --- |
| **LOD** |  | 79 | 0 | 2 | 4 | 26 | 599 | 2 | 4 | 1 | 0 | 6 |
| Young Leaf | 1 | <LOD | 6332 | 15266 | 8522 | 1545 | 4732 | 34 | 199 | 2 | 29 | 71 |
| Old Leaf | 1 | 202 | 9380 | 1831 | 14882 | 285 | 1455 | 84 | 331 | 1 | 7 | 54 |
| Stem | 1 | 310 | 3111 | 2563 | 3594 | 189 | 2231 | 7 | 172 | <LOD | 4 | 13 |
| Root | 1 | 620 | 2288 | 2993 | 4504 | 280 | 1518 | 133 | 116 | 2 | 5 | 64 |
| Seedcoat | 1 | <LOD | 1763 | 10019 | 2159 | 553 | 1172 | 17 | 64 | 2 | 6 | 22 |
| Seed | 1 | <LOD | 1457 | 10218 | 3302 | 563 | 800 | 24 | 51 | 2 | 5 | 13 |
| Young Leaf | 2 | 286 | 6969 | 3177 | 10422 | 316 | 1219 | 50 | 209 | <LOD | 7 | 38 |
| Old Leaf | 2 | 230 | 3895 | 3007 | 11149 | 117 | 1528 | 69 | 129 | <LOD | 7 | 41 |
| Root | 22 | 168 | 3805 | 3121 | 11653 | 154 | 1167 | 117 | 179 | 1 | 4 | 106 |
| Young Leaf | 3 | 93 | 7714 | 3105 | 7247 | 126 | 1002 | 41 | 224 | <LOD | 12 | 57 |
| Stem | 3 | 189 | 5100 | 6489 | 4564 | 126 | 1171 | 28 | 535 | <LOD | 3 | 13 |
| Old Leaf | 4 | 727 | 3039 | 2174 | 8475 | 270 | 1124 | 92 | 134 | <LOD | 4 | 48 |
| Root | 4 | 458 | 1609 | 4369 | 10736 | 267 | 952 | 101 | 63 | <LOD | 3 | 123 |
